# A hierarchical spatial assembly approach of silica-polymer composites leads to versatile silica/carbon nanoparticles

**DOI:** 10.1126/sciadv.adi7502

**Published:** 2023-10-04

**Authors:** Dan Cheng, Jun Zhang, Jianye Fu, Hao Song, Chengzhong Yu

**Affiliations:** ^1^ Australian Institute for Bioengineering and Nanotechnology, The University of Queensland, Brisbane, QLD 4072, Australia.; ^2^ College of Chemistry and Chemical Engineering, China University of Petroleum, Qingdao 266555, China.

## Abstract

Assembly of silica and polymer in the absence of surfactant templates is an emerging strategy to construct intricate nanostructures, whereas the underlying mechanism and structural versatility remain largely unexplored. We report a hierarchical spatial assembly strategy of silica-polymer composites to produce silica and carbon nanoparticles with unprecedented structures. The assembly hierarchy involves a higher length scale asymmetric A-B-A core-shell–type spatial assembly in a composite sphere, and a nanoscale assembly in the middle layer B in which the silica/polymer ratio governs the assembled structures of silica nanodomains. Through an in-depth understanding of the hierarchical spatial assembly mechanism, a series of silica and carbon nanoparticles with intriguing and controllable architectures are obtained that cannot be easily achieved via conventional surfactant-templating approaches. This work opens an avenue toward the designed synthesis of nanoparticles with precisely regulated structures.

## Teaser

Versatile silica/carbon nanoparticles are obtained via a hierarchical spatial assembly strategy of silica-polymer composites.

## INTRODUCTION

Self-assembly is a versatile approach to organizing matters into well-defined structures at various length scales (*1*). A typical example is the self-assembly of amphiphilic surfactant molecules into micelle (*2*–*4*), vesicle (*5*), and liquid crystals (*6*), which can be used as templates to synthesize nanoporous materials with diverse compositions (e.g., siliceous and carbonaceous), mesostructures, and morphologies (*7*–*10*). Originating from the classical Stöber method to produce silica (*11*) and polymeric nanoparticles (*12*, *13*), surfactant-free silica and polymer assembly has attracted increasing attention in recent years. Silica and polymer can self-organize into microscale objects (*14*–*16*). At another dimension, silica-polymer assembly may occur at the nanoscale (*17*–*19*). This approach has been applied to prepare both silica and carbon nanoparticles with unique structures, e.g., silica nanoparticles with rambutan-like surface roughness (*20*, *21*) and particle asymmetry (*22*), and carbon nanoparticles with invaginated structures (*23*, *24*), hollow cavities (*25*), tunable pore sizes (*18*), and pore structures (*26*).

It should be pointed out that the surfactant self-assembly fundamentals are well-established (*7*, *27*). As a typical example, the advance from conventional liquid crystal templating (*28*, *29*) to micelle templating has guided the synthesis of virus-like silica nanoparticles (*30*), core-satellite type silica nanoparticles (*31*), and hollow mesoporous carbon nanoparticles with an ultrathin monolayer of spherical pores (*32*). However, the discovery of surfactant-free silica-polymer assembly is still in its infancy. There is a lack of deep understanding of the self-assembly principles in this system. It also remains challenging to design silica nanoparticles with controllable shapes and dimensions of nanotopography, or carbon nanoparticles with adjustable surface roughness through current silica-polymer assembly approaches, which are beneficial for the ever-increasing demands for silica and carbon nanoparticles in various applications and diverse fields (*33*–*36*).

**Fig. 1.**
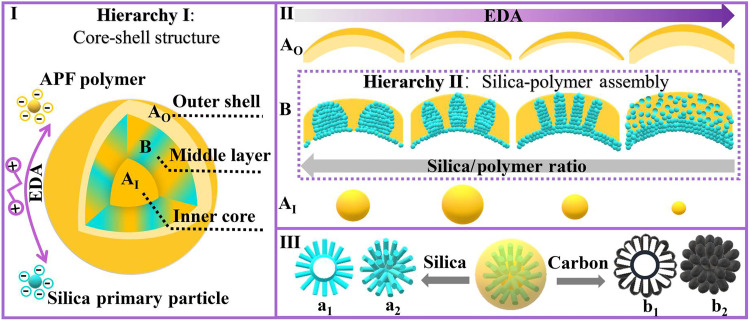
Schematic illustration of the HSA mechanism of silica-polymer composites. (**I**) The first level of the hierarchy is the spatially assembled A_I_-B-A_O_ core-shell–type architecture consisting of a polymer inner core (A_I_), a middle layer of SPC (B), and a polymer outer shell (A_O_). (**II**) The diameter of A_I_ in the inner core, the silica-polymer assembly in B (the second level of the hierarchy), and the thickness of the outer shell A_O_ are dependent on the ethylene diamine (EDA) concentration. (**III**) The SPCs derived from HSA lead to nanoparticles with adjustable structures, e.g., silica (blue) with rodlike topography and tunable aspect ratios (a_1_ and a_2_) and carbon (black) nanoparticles with varied channel sizes and surface roughness (b_1_ and b_2_).

Here, a hierarchical spatial assembly (HSA) strategy of silica-polymer composites (SPCs) is reported. As shown in Fig. 1-I, the first level of the hierarchy is an asymmetric “A_I_-B-A_O_” core-shell–type spatial assembly of SPCs in a sphere, including an 3-aminophenol- formaldehyde (APF) polymer inner core (A_I_), a middle layer of SPC (B), and a polymer outer shell (A_O_). Ethylene diamine (EDA) used in the synthesis affects not only the sizes of the inner core (A_I_) and the thickness of the outer shell (A_O_) but also the second level of the hierarchy, e.g., the silica-polymer assembly in the middle layer related to the silica/polymer ratio (Fig. 1-II). At low EDA concentrations, spherical and ellipsoidal silica nanodomains with high silica-polymer ratios are formed because of the kinetically favored growth of silica. With increased EDA amount and enhanced silica-polymer interaction, the silica/polymer ratio is decreased, leading to the formation of rodlike and eventually disordered silica nanodomains, a behavior analogous to surfactant assembly. Moreover, both levels of the hierarchy determine the solid/hollow cavity and rough/smooth surface of carbon nanoparticles obtained by carbonization and silica removal. Consequently, a selection of silica and carbon nanoparticles is obtained (Fig. 1-III), such as hollow silica nanospheres with adjustable aspect ratios of rodlike domains (a_1_ and a_2_) and hollow carbon nanoparticles with controllable pore channel sizes and surface roughness (b_1_ and b_2_).

## RESULTS

To demonstrate the HSA strategy, a systematic study is crucial. Therefore, this work will not be introduced by choosing the most attractive samples. Instead, the structural changes of SPCs, silica, and carbon nanoparticles in a large range of EDA amount variation will be described first. Such information, together with further investigation of the silica and carbon nanoparticles using electron tomography (ET), will provide evidence of our proposed mechanism before moving to Discussion with additional information. A proof-of-concept demonstration, e.g., the impact of silica nanoparticle structure on DNA delivery, will also be briefly presented.

### Structure characterization of silica-polymer composites

The A_I_-B-A_O_ core-shell–type SPCs were assembled in alkaline ethanol-water solutions, using tetraethyl orthosilicate (TEOS) and 3-aminophenol/formaldehyde (3-AP/F) as silica and polymer precursors, respectively (see details in Materials and Method and fig. S1). EDA was used to regulate the silica-polymer assembly, and a series of SPC-*x* (*x* = 1 to 8, corresponding to the EDA amount of 0.08, 0.10, 0.12, 0.15, 0.22, 0.29, 0.31, and 0.51 ml, respectively) were prepared. Transmission electron microscopy (TEM) images of SPC-*x* are presented in fig. S2 (see the Supplementary Materials), all showing a spherical morphology. Notably, varied contrast (silica exhibits a darker contrast than polymer) within a single nanoparticle was observed in all samples, indicating the incorporation of silica and polymer in the composites.

**Fig. 2.**
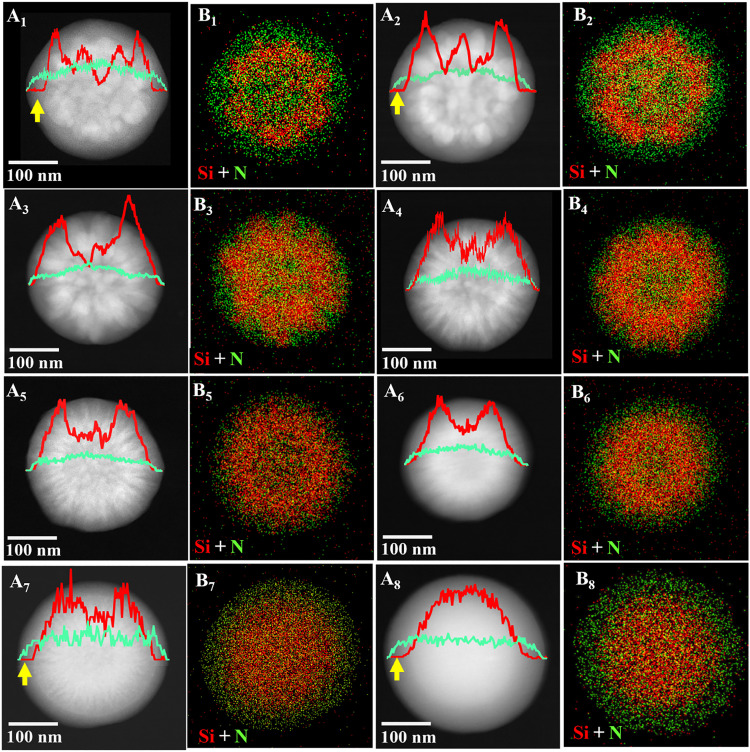
Structural and compositional characterization of silica-polymer composites. (**A**_**1**_ to **A**_**8**_) HAADF-STEM images and (**B**_**1**_ to **B**_**8**_) elemental mapping images of SPC-*x*. The elemental mapping images are the overlap of Si (in red) and N (in green). The line scan elemental distribution of Si and N is also shown in the inset of (A_1_) to (A_8_).

To characterize the spatial distribution of silica and polymer in the composite, high-angle annular dark-field scanning TEM (HAADF-STEM) and corresponding elemental analysis through mapping and line scans were recorded for SPC-*x*. As shown in Fig. 2, the contrast difference in the STEM images (Fig. 2, A_1_ to A_8_) and the coexistence of silicon (red)/nitrogen (green) provide evidence of the composition distribution (Fig. 2, B_1_ to B_8_). From outside to the center of one individual nanoparticle, SPC-1 and SPC-2 prepared at a relatively low EDA amount show an obvious polymer-rich outer shell (evidenced by a lower contrast). This is also evident in the line scan elemental distribution curves (indicated by yellow arrows). By increasing the EDA amount, the outer polymer shell becomes thinner and even not obvious for SPC-3 to SPC-6, and then reappears in SPC-7 and SPC-8. Judging from silicon line scan profiles that show a valley inside the nanoparticle, a polymer-rich core is evident in all samples except SPC-8. In between the polymer-rich core and outer layer, there is a middle layer with overlapped silicon and polymer. However, their fine structures are difficult to be identified. Therefore, carbon and silica nanoparticles will be investigated separately in the following sections to better understand the composite structures.

**Fig. 3.**
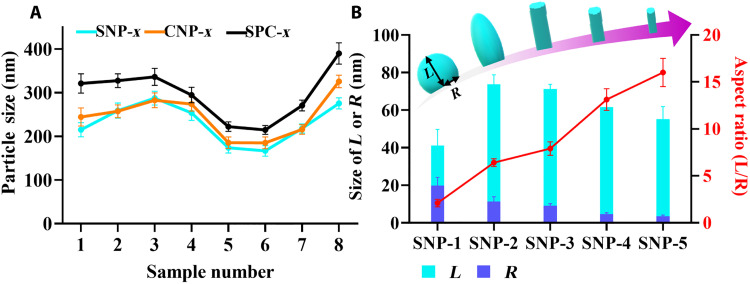
Dimensional characterization. (**A**) The particle sizes of SNP-*x*, CNP-*x*, and SPC-*x* (*x* = 1 to 8). (**B**) The sizes of length (*L*) and radius (*R*) and the aspect ratio of spherical (SNP-1), ellipsoidal (SNP-2), and one-dimensional (1D) rodlike (SNP-3 to -5) silica subunits.

The particle sizes of SPC-*x* were also measured from at least 50 nanoparticles from TEM images via ImageJ software. As shown in Fig. 3A (black curve), the particle size firstly increases from 321.3 (SPC-1) to 336.3 nm (SPC-3), and then decreases to 214.7 nm (SPC-6). The size sharply increases to 270.5 nm (SPC-7) and continues increasing to 389.9 nm (SPC-8).

### Structure characterization of silica nanoparticles

**Fig. 4.**
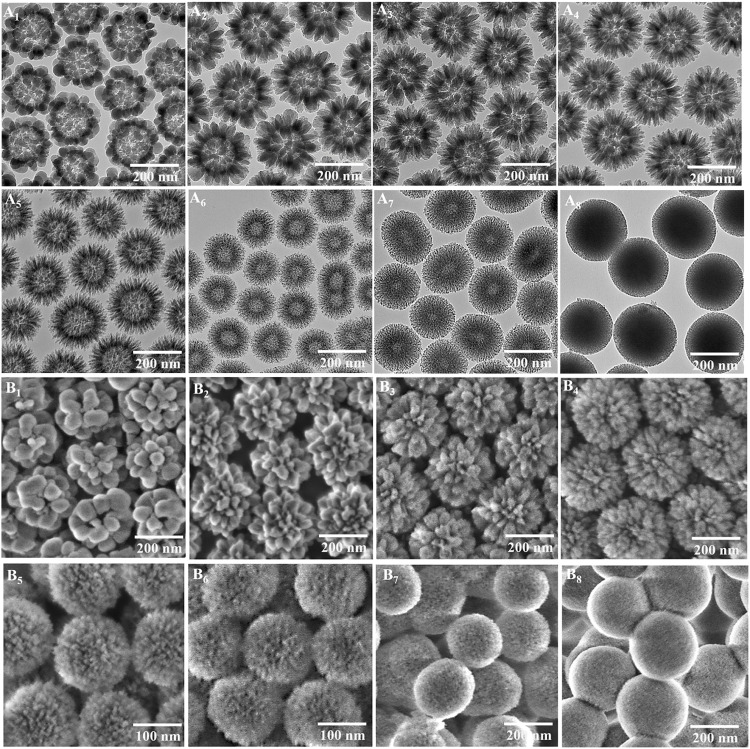
Morphological characteristics of silica nanoparticles. TEM images (**A**_**1**_ to **A**_**8**_) and SEM images (**B**_**1**_ to **B**_**8**_) of SNP-*x* (*x* = 1 to 8) with spherical (*x* = 1), ellipsoidal (*x* = 2), 1D rodlike (*x* = 3 to 6) surface topography and disordered nanoporous network (*x* = 7 to 8).

Silica nanoparticles (SNP-*x*) were obtained via calcination of SPC-*x* in air to remove the polymer, with their TEM images shown in Fig. 4 (A_1_ to A_8_). SNP-1 to -7 all clearly show hollow cavities, indicating the existence of polymer cores in the composites before calcination. With the increase of EDA amount, the surface nanotopography of SPC-*x* evolves from spherical (SNP-1) to ellipsoidal (SNP-2) and rodlike (SNP-3 to -6) shapes. For SNP-7 and SNP-8, the nanotopography is hard to observe under TEM, instead disordered nanoporous networks are evident. SNP-8 appears as a dense silica nanosphere without a hollow interior; however, the porous structure can still be identified from the TEM image at high magnification (fig. S3). The evolution of surface nanotopography can be clearly identified from scanning electron microscopy (SEM) images as shown in Fig. 4 (B_1_ to B_8_). Apart from SNP-8 showing a smooth surface, SNP-1 to -7 samples all exhibit a rough texture with evident transitions from roundish nanodomains to elongated subunits with gradually decreased diameters with increased EDA amount, in accordance with TEM observations.

The particle sizes of SNP-*x* were also measured. As shown in Fig. 3A (blue curve), with increased EDA amount, the particle size of SNP-*x* firstly increases from 215.2 (SNP-1) to 287.5 nm (SNP-3), and then decreases down to 166.7 nm (SNP-6). Afterward, the particle size rapidly increases to 218.2 nm for SNP-7 and 275.3 nm for SNP-8. The trend of size change of SNP-*x* is similar to SPC-*x.* Moreover, the aspect ratio of the silica subunits in SNP-1 to -5 was measured, defined by the average length (*L*) over radius (*R*) of 50 subunits identified from TEM images. These parameters are difficult to measure from the TEM images of other samples. As shown in Fig. 3B, the length firstly increases from SNP-1 (41.2 nm) to SNP-2 (73.7 nm) and then decreases from SNP-3 (71.2 nm) to SNP-5 (55.3 nm). Instead, the radius continuously decreases from 19.8 nm of SNP-1 to 3.5 nm of SNP-5. From the measured *L* and *R* values, the aspect ratio of the silica subunit is calculated to be 2.1 and 6.4 for SNP-1 and SNP-2, respectively, consistent with their spherical and ellipsoidal structural feature. For SNP-3 to -5, with increased EDA amount, the aspect ratio increases to 7.9, 13.1, and 16.0, respectively.

The porous properties of SNP-*x* were characterized via N_2_ sorption analysis. The adsorption/desorption isotherms and pore size distribution (PSD) curves calculated from the adsorption branch using the Barrett-Joyner-Halenda (BJH) model are shown in fig. S4. The Brunauer-Emmett-Teller (BET)–specific surface area, average pore size, and total pore volume are summarized in table S1. With increased EDA amount, the specific surface area of SNP-*x* increases continuously from 50.1 (SNP-1) to 672.7 m^2^/g (SNP-8). An obvious peak at ~130 nm in the PSD curves of SNP-1 could be attributed to the voids from the irregular stacking of nanoparticles. This peak is not obvious for SNP-2 and -3. The pore size of SNP-4 is ~50.0 nm, and then decreases in the rest samples. SNP-8 has the smallest pore size of 1.6 nm which falls into the micropore realm. However, the change in total pore volume has no clear trend. This is possible because the volume adsorbed in the interparticle packing voids at a relative pressure (*P*/*P*_0_) higher than 0.95 (*37*, *38*) varies differently (e.g., SNP-7 and -8), or could not be easily differentiated from large pores (e.g., SNP-1, -2, and -3).

### Characterization of carbon nanoparticles

**Fig. 5.**
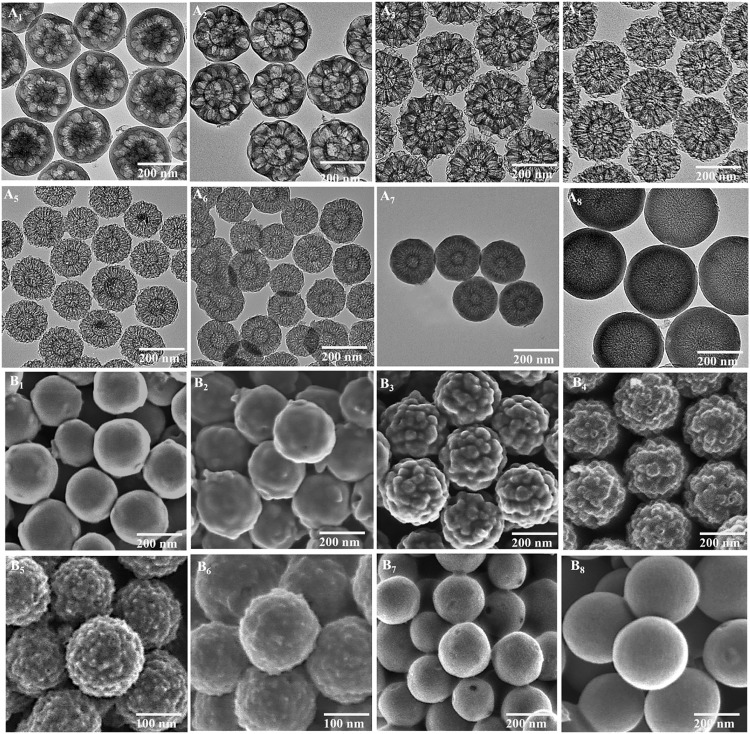
Structure characteristics of carbon nanoparticles. TEM (**A**_**1**_ to **A**_**8**_) and SEM images (**B**_**1**_ to **B**_**8**_) of CNP-*x* (*x* = 1 to 8)

Carbon nanoparticles (CNP-*x*) were obtained via carbonization of SPC-*x* in N_2_ and subsequent removal of silica. Combining the TEM and SEM observations (Fig. 5), CNP-1 has a solid inner core (Fig. 5A_1_), a porous middle layer with spherical voids, and an outer shell that is dense and smooth (Fig. 5B_1_). For CNP-2 to -7, a hollow inner cavity is observed with gradually decreased size, but not evident in CNP-8 (Fig. 5, A_2_ to A_8_). The pore shape in the middle porous layer is more complicated in CNP-2 to -8 and cannot be easily judged from TEM. Besides, the thickness of the outer carbon shell decreases in CNP-2 than in CNP-1 but increases in CNP-7 to -8. However, the outer carbon shell is hardly observed in CNP-3 to -6, sharing a similar trend to the change of polymer shell (A_2_) in SPC-*x*. The surface morphology of CNP-*x* changes from the smooth surface of CNP-1 and CNP-2 to rough surfaces in CNP-3 to -6, and then back to a relatively smooth surface in CNP-7 to -8 (Fig. 5, B_1_ to B_8_). The particle size of CNP-*x* is shown in Fig. 3A (orange curve) to allow a direct comparison with SPC*-x* and SNP-*x*. The overall particle size variation trend with increasing EDA amount is similar in the three series of samples.

The N_2_ adsorption/desorption isotherms and corresponding PSD curves of CNP-*x* are shown in fig. S5, from which the measured BET-specific surface area, pore size, and total pore volume are listed in table S2. With increased EDA amount, the specific surface area increases from 216.6 (CNP-1) to 1304.2 m^2^/g (CNP-*7*), and then decreases to 547.2 m^2^/g (CNP-8). CNP-1 shows a broad PSD ranging from 28.2 to 103.1 nm (centered at 50.4 nm). For CNP-2 to -8, the pore sizes continuously decrease from 50.0 to 2.4 nm. Similar to their silica counterparts, the changes in total pore volumes have no obvious trend. All CNP-*x* samples show a steep desorption branch with a lower closure point of hysteresis located around *P*/*P*_0_ of 0.47, indicative of cavitation-induced evaporation (*39*). For CNP-2, -3, and -4, the observation of another capillary evaporation at higher *P*/*P*_0_ of ~0.7 to 0.8 indicates the existence of open large pores (*40*).

The chemical composition of selected carbon nanoparticles (CNP-1, -4, and -7) was measured by x-ray photoelectron spectroscopy (XPS). As shown in fig. S6A, the XPS spectra of CNP-1, -4, and -7 all show three typical peaks for C 1s, N 1s, and O 1s with the N content increased from 3.63 (CNP-1) to 5.43 atomic % (at %) (CNP-*7*). The increased N content is in accordance with the increased amount of EDA used in the synthesis that may be incorporated into the polymer framework (fig. S1). The N 1s spectra of all CNP-1, -4, and -7 can be deconvoluted into three single peaks (fig. S6B), which are correlated to pyridinic N (398.2 eV), pyrrolic N (400.7 eV), and oxidized N (402.4 eV) (*23*).

### Electron tomography characterization

**Fig. 6.**
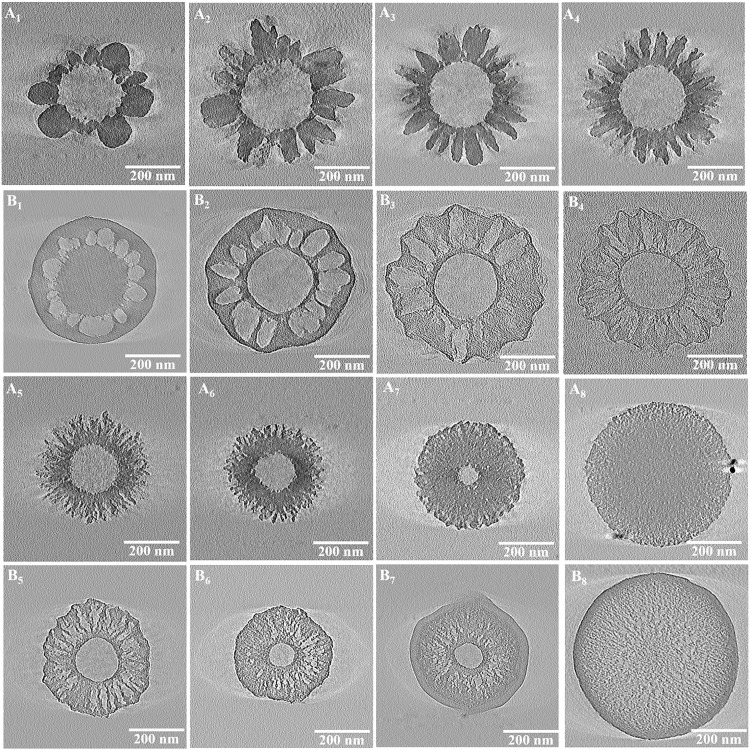
Precise characterization of nanoparticle inner structures. ET slices of silica nanoparticles [SNP-*x*, (**A**_**1**_ to **A**_**8**_)] and carbon nanoparticles [CNP-*x*, (**B**_**1**_ to **B**_**8**_)].

To clearly characterize the complex structures of silica and carbon nanoparticles, ET was used (*41*–*43*). Tomograms sliced through the center of a single nanoparticle of both SNP-*x* and CNP-*x* were illustrated in Fig. 6 (A_1_ to A_8_ and B_1_ to B_8_, respectively). In Fig. 6, SNP-*x* and CNP-*x* obtained at the same EDA amount (e.g., with the same *x*) were displayed in a top-down format to reflect the silica-polymer assembly in SPC-*x*. As a typical example, the ET slice of SNP-1 (Fig. 6A_1_) shows a hollow cavity and spherical silica subunits closely packed into a shell. From the ET slice of CNP-1 (Fig. 6B_1_), a solid core, a thick outer shell with smooth contour, and a middle porous layer with spherical large pores separating the core and shell can be observed. The complementary information from the ET slices of SNP-1 and CNP-1 provides a clear picture of the A_I_-B-A_O_ assembly in SPC-1 compared to that obtained from TEM and SEM results.

For the rest SNP-*x* samples, with increased EDA amount the hollow cavity size decreases, eventually, SNP-8 has an overall solid structure. The shape and size of silica subunits are clearly seen from the ET slices. In general, the silica subunits experience a spherical to ellipsoidal and rodlike structural evolution (SNP-1 and SNP-3), and then the rodlike subunits decrease in both diameter and length until SNP-6. Note that there is a dense silica transition layer between the hollow inner cavity and the surface silica subunits with increased thickness from SPC-2 to -7 (Fig. 6, A_2_ to A_7_), with the disappearance of the hollow inner cavity in SNP-8. This dense silica layer is essential to stabilize the integrity of individual silica subunits distributed above the hollow inner cavity.

For CNP-*x* samples, it is unexpected that CNP-2 to -7 present a hollow cavity, different from CNP-1, indicating the solid polymer core (A_1_) in SPC-*x* may undergo different behavior during carbonization (see details in Discussion). Compared to CNP-1, the thickness of the outmost shell reduces in CNP-2, which is the thinnest in CNP-3 to CNP-6, and then becomes thick again in CNP-7 and CNP-8. The information on pore shapes and sizes in CNP-*x* samples is clearer in ET slices than in TEM. In particular, when comparing the ET slices of SNP-*x* and CNP-*x* from the same SPC-*x* sample, it is concluded that the silica subunits in SNP-*x* are porogens and determine the pore structure of CNP-*x* (*x* = 1 to 6). For CNP-7 and CNP-8, the silica subunits are less organized in the corresponding composites (SPC-7 and SPC-8), thus carbon nanoporous spheres with disordered pore arrangements are obtained. The above results also indicate that the simultaneous change in the silica subunits and the outer shell thickness in SPC-*x* is responsible for the surface roughness of CNP-*x* samples (see Discussion).

### The impact of EDA amount on the inner core (A_I_) formation

The first step in the HSA strategy is the polymer inner core (A_I_) formation (Fig. 1 and fig. S1). In the synthesis of SPC-*x*, the APF core was allowed to polymerize for 30 min before the addition of silica precursors (see the "Fabrication of silica-polymer composites" section in Material and Methods and fig. S1). Because the polymer core is hidden inside SPC-*x*, it is difficult to accurately measure its size. Alternatively, the hollow cavity in SNP-*x* is generated after removing the polymer core via calcination, thus the hollow cavity size change can be used as an approximate measure of the polymer core size change (at least the trend). As shown in fig. S7 (blue curve), the average diameter of the hollow cavity in SNP-*x* increases from 126.0 (SNP-1) to 137.6 nm (SNP-2), and then continuously decreases to 46.5 nm (SNP-7).

To directly observe the impact of EDA amount on the polymer core formation and avoid the influence of silica or calcination treatment, the APF polymer particles at different EDA amounts (PC-*x*) after 30 min of polymerization were collected for TEM observation (fig. S8, A_1_ to A_8_), without further introducing silica precursors. PC-1 to -7 show a solid spherical morphology with their sizes decreased with EDA amount (fig. S8, A_1_ to A_7_), while fragments with irregular morphology were observed in PC-8 (fig. S8A_8_) at the highest amount of EDA. The particle sizes of PC-*x* measured from TEM images show the same trend (fig. S7, black curve) as the hollow cavity size change in SNP-*x* (fig. S7, blue curve). For the sample obtained at the same EDA amount (*x*), the hollow cavity size in SNP-*x* is smaller than the particle size of PC-*x*, due to calcination-induced contraction. Besides, the sizes of PC-*x* were also characterized by dynamic light scattering (DLS; fig. S7, gray curve), showing a similar trend to that measured from TEM except overall larger sizes due to the surface hydration by water molecules (*44*).

To understand the influence of EDA on the size change of APF, another sample PC-0 was prepared without adding EDA. As shown in fig. S7, the size of PC-0 is smaller compared with that of PC-1 and -2. It is suggested that EDA initially promotes the APF polymerization and particle growth into larger sizes (from PC-0 to -2). Further increasing EDA amount leads to continuously decreased particle sizes from PC-3 to -7, mainly due to the delayed APF polymerization caused by depletion of formaldehyde at excessive EDA amounts via Schiff base reaction (*23*). The delayed APF polymerization at high EDA amounts is also indirectly evidenced by the color change as shown in fig. S9. A similar phenomenon has been reported in a resorcinol-formaldehyde (RF) system, where the particle size of RF spheres decreases with increasing EDA amount, and there is no particle formation at the highest amount of EDA used in this study (*45*).

### The impact of EDA amount on the silica/polymer ratio in the silica-polymer composite (A_I_-B-A_O_) and middle layer (B)

**Fig. 7.**
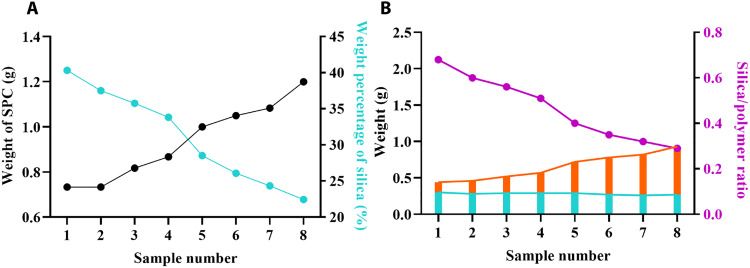
Compositional analysis of the silica-polymer composites. (**A**) The total weight of SPC-*x* (*x* = 1 to 8, black curve) and the weight percentage of silica (blue curve) characterized by thermogravimetric analysis (fig. S10). (**B**) The weight of silica (blue curve/bar), polymer (orange curve/bar), and the ratio of silica to polymer in the composite (purple line).

Increasing the EDA amount affects not only APF polymerization on the polymer core sizes but also the silica-polymer assembly behavior in the middle layer (B). To understand how the EDA amount affects the assembled structure in layer B, the total weight of SPCs (SPC-*x*) was first recorded, which increased continuously from 0.73 g in SPC-1 to 1.20 g in SPC-8 (Fig. 7A, black curve). Then, the silica weight percentage in the SPC-*x* was quantified via thermogravimetric analysis (fig. S10). As summarized in Fig. 7A (blue curve), the weight percentage of silica decreased consistently from SPC-1 (40.3%) to SPC-8 (22.4%).

The weight of silica in the SPC-*x* can be calculated by multiplying the silica weight percentage by the total weight. As shown in Fig. 7B, the weight of silica (blue) is nearly constant (~0.28 g) in all eight samples, close to the theoretical silica weight calculated by the feeding amount of TEOS. This observation indicates that, at varied EDA amounts, the silica precursor was almost fully incorporated into the SPC-*x*; however, the polymer content (orange) increased. This leads to the continuously decreased silica/polymer ratio (purple line) in the SPC (A_I_-B-A_O_) with increasing EDA amount. The ratio of silica to polymer in the middle layer (B) is difficult to calculate because it is hard to differentiate the sizes of three parts (A_I_, B, and A_O_) individually in SPC-*x* nor in SNP-*x* (A_O_ is removed) or CNP-*x* (the polymer contract behavior in three parts may be different). Nevertheless, it can be estimated that the silica/polymer ratio in the middle layer of SPC-1 to SPC-2 should be increased than that measured from SPC-1 to SPC-2 when both the polymer inner core and outer shell (A_I_ and A_O_) are considered. This increase is expected to be not significant in other SPC-*x* samples due to gradually reduced inner core sizes (there is an obvious core in SPC-8) or negligible shell (e.g., SPC-3 to -6). Therefore, the overall trend of the silica/polymer ratio in the middle layer is expected to be similar to that in the SPC-*x* composites.

Note that the increased polymer content with increasing EDA amount in SPC-*x* is different from the observation in PC-*x* where the APF polymerization is retarded. In the synthesis of SPC-*x*, extra 3-AP, and TEOS were introduced with a further reaction time of 3 hours. To allow a direct comparison, two samples were prepared, one is SPC-8 and another is a control sample without adding TEOS in the second step, while other reaction conditions are the same as SPC-8. Colloidal particles were obtained in both reactive solutions after 3 hours (fig. S11A). Compared to the particle size of SPC-8 (389.9 nm; fig. S11B), the size of the control sample is nearly five times larger (~1937 nm; fig. S11C) with a yield (~0.82 g) similar to the net polymer weight in SPC-8 (~0.93 g; Fig. 7A). This observation provides two hints: (i) Further addition of 3-AP and prolonged reaction time (3 hours) promote the APF polymerization, mainly due to the reversible Schiff base reaction and (ii) the introduced silica species interact with APF and induce the silica-polymer assembly in the middle layer (B).

### The impact of EDA amount on the thickness of polymer outer shell (A_O_)

In addition to the formation of polymer core (A_I_) and silica-polymer middle layer (B), the amount of EDA also has impact on the formation of the polymer outer layer (A_O_). Judged from both Fig. 2 (SPC-*x*) and Fig. 5 (CNP-*x*, B_1_-B_8_), the polymer outer layer is more obvious at low and high EDA amount (SPC-1, -2, -7, and -8), but not in the middle range (SPC-3 to -6). At low EDA amounts (*x* = 1, 2), the silica to polymer weight ratios are the highest among all SPC-*x* (Fig. 7B), indicating the deposition of a silica-rich middle layer followed by the growth of the outer polymer-rich shell. For SPC-3 to -6, the absence of the polymer outer layer suggests that all the polymer and silica co-assemble in the middle layer. At higher EDA amounts (*x* = 7, 8), the observation of the polymer outer layer in SPC-7 and -8 may be explained by the size reduction of the polymer core (not obvious in SPC-8), leading to excessive polymer precursors after silica-polymer co-assembly that form a polymer out layer.

## DISCUSSION

In this study, we have synthesized and characterized a series of SPC-*x* and corresponding silica (SNP-*x*) and carbon (CNP-*x*) samples. From the impact of EDA amount on the formation of the polymer inner core (A_I_), the middle SPC layer (B), and the polymer outer shell (A_O_), a formation mechanism can be proposed as follows.

### Silica/polymer ratio governs silica assembly: An analog to surfactant assembly

The self-assembly of silica-polymer is governed by the silica/polymer ratio (Fig. 1-II), specifically for the structure in the composite middle layer (B) because the inner core (A_I_) and the outer shell (A_O_) are basically polymers. It is suggested that the proposed HSA mechanism is analogous to the well-established surfactant assembly in aqueous solutions (*46*, *47*) or surfactant-templated mesostructured silicate materials (*7*). After the formation of the inner polymer core (A_I_; fig. S1), the hydrolysis and condensation of TEOS result in the generation of silica primary particles (SPP; fig. S12) (*48*–*50*). Because of the reversible exchange between hydroxyl (Si-OH) and ethoxy (Si-OEt) groups under alkaline water-ethanol solutions (*48*), it is expected that SPP have an amphiphilic nature (analogous to surfactant molecules) and experience continuous condensation and association into silica assemblies with larger sizes (analogous to micelles) (*48*). Nevertheless, the specific shape of silica assembly in the silica-polymer system is determined by the silica/polymer ratio. In dilute solutions, micelles (*51*, *52*) or disordered mesostructured materials (*53*–*55*) are formed. This is a similar observation in SPC-7 to -8, where the silica/polymer ratio is lower compared to other SPC-*x*; consequently, silica is dispersed in a disorganized manner in the continuous polymer matrix. With an increased silica/polymer ratio, SPP organize into oriented one-dimensional (1D) rodlike nanostructures surrounded by discontinuous polymer phases (e.g., SPC-3 to -6), an analog to the formation of rodlike micelles or mesostructured silicate materials with 1D pore channels when surfactant concentration is higher than CMC.

It is noted that traditional mesoporous materials obtained via surfactant/silica assembly usually have uniform and narrow PSD (*9*). However, the PSD of SNP obtained via silica-polymer self-assembly is generally broader (fig. S4). This is possible because the SPP and their aggregates are not that uniform compared to surfactants and micelles. Moreover, the mesopores in traditional mesoporous materials are generated after the removal of surfactant templates. However, in the silica-polymer assembly system, the mesopores are continuous after the removal of the polymer phase, surrounded by silica subunits that are less ordered than surfactant assemblies. In addition, compared with dendritic mesoporous silica nanoparticles (DMSN) obtained via surfactant-silica assembly (*56*, *57*), both large-pore DMSN and SNP have radial pores and relatively broad PSD. However, their pore wall structures are different. The wall in large-pore DMSN is usually formed by sheet-like silica structures and the pores are isolated, while, in SNP, the silica wall can be formed by rodlike subunit and the pore space is continuous.

### EDA as a linker to adjust silica-polymer interaction

The use of EDA is crucial in adjusting the interfacial nucleation and the structure in the middle layer B. As shown in Fig. 1 and fig. S1, both the SPP and APF are negatively charged because of deprotonation of hydroxyl groups, thus mutually repulsive. This is evidenced by the formation of silica and polymer spheres that are separated in the absence of EDA (fig. S13). Besides, the surface charge of APF sphere fabricated using ammonia hydroxide as the catalyst was −29.5 mV (fig. S14) (*58*). After adding EDA with amounts equivalent to the synthesis of PC-1 to -8, the surface charge of APF sphere generally increased with the EDA amount. This indicates that EDA is positively charged under our synthesis conditions and attaches to the surface of APF polymer to partially neutralize the negative charge. For EDA with two amine groups that can be protonated, it is suggested that another end of EDA is also positively charged that can further attract the negatively charged SPP, thus EDA serves as the linker to bind silica and APF polymer.

### EDA amount adjusts the silica/polymer ratio and the assembled structures

The proposed mechanism explains the impact of EDA amount on the assembled structures of SPC-*x*. When the EDA amount is low (e.g., SPC-1), the low concentration of EDA at the interface of preformed APF core (A_I_), e.g., weak interfacial interaction, induces the heterogeneous nucleation of silica with a low density after introducing the silica source. This leads to the kinetically favored silica growth at an early stage (fig. S15, A_1_ and A_2_). The APF polymer may fill into the voids between silica islands at a later stage (fig. S15, A_3_ and A_4_). With little spatial limitation from polymer as evidenced by the final high silica/polymer ratio in SPC-1, SPP prefers to grow into spherical morphology to lower surface energy. HAADF-STEM and elemental mapping images further support the above conclusion (fig. S16).

With increased EDA concentration, a more positively charged APF core promotes heterogeneous nucleation of silica with a higher density as well as a smaller size (e.g., in SPC-4; figs. S15B and S16B), leading to decreased surface roughness and eventually relative smooth surface (e.g., in SPC-7; figs. S15C and S16C). Moreover, the co-assembly of SPP and APF occurs at an earlier time point (e.g., 2 min) as judged by the structural difference between SPC-1 and SPC-4/7. Therefore, the assembly behavior of SPP in the middle layer B is strongly affected by the relative silica/polymer ratio. When SPP is in high concentration (e.g., SPC-3 to -6), a rodlike morphology is formed because SPP prefers to add on the tip of existing silica islands (Fig. 1). Further increasing the EDA concentration leads to even stronger interaction between SPP and polymer, thus the formation of silica with small domain sizes surrounded by APF, which further aggregate into disordered structures in the composite layer B in SPC-7 (and -8) samples.

### Understanding the A_I_-B-A_O_ structures on the hierarchical structures of carbon

**Fig. 8.**
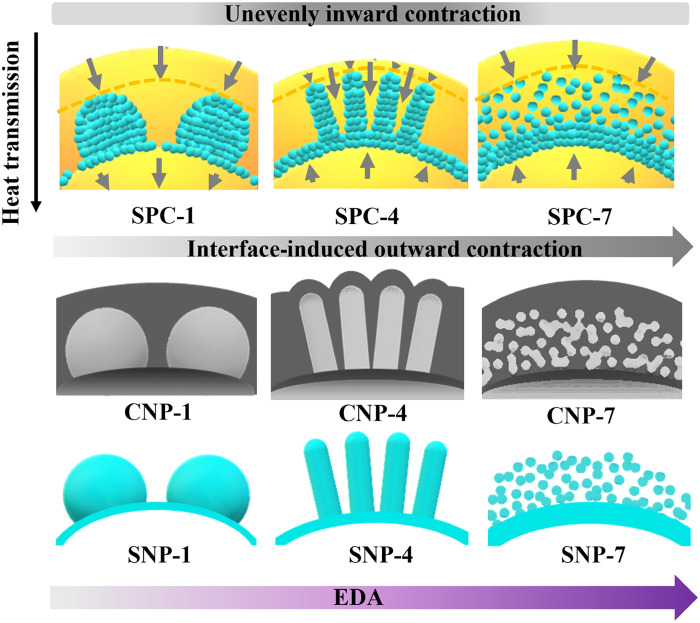
Schematic illustration of the formation mechanism of CNP-1, -4, and -7. The different inner structures (e.g., solid core or hollow cavity), pore shapes, and outer surface roughness of CNP-1, -4, and -7 are related to the core-shell structures of SPC-1, -4, and -7 that are dependent on the amount of EDA.

Understanding the A_I_-B-A_O_ assembly in SPC-*x* is important to explain the versatile hierarchical structures of CNP-*x*. As shown in Fig. 8, the structures of CNP-1, CNP-4, and CNP-*7* are determined by the corresponding SPC-*x* samples. For SPC-1 and SPC-7 with thick polymer outer shell (A_O_), CNP-1 and CNP-7 with smooth outer surface are formed because of even contraction of the polymer during carbonization. When the outer shell is thin (e.g., SPC-4), the contraction is uneven, which is hindered at the rigid silica interface. This leads to the formation of a rough surface in CNP-4, and the nanoscale roughness is determined by the diameter of rodlike silica subunits, as reflected in CNP-3, -4, -5, and -6 samples. Besides, the pore shape of CNP-1, -4, and -7 corresponds to their silica counterparts (SNP-1, -4, and -7) because silica serves as the porogen.

The polymer inner core (A_I_) in SPC-*x* has two contract behaviors, also determined by the EDA amount. For SPC-1 prepared with the least amount of EDA, the thickness of the silica layer coated on the polymer core is the smallest, while the rest SPC-*x* samples (except SPC-8) have a thicker silica layer as demonstrated in the ET slides of silica (Fig. 6, A_1_ to A_8_). A thick silica layer may provide a rigid interface and cause outward contraction of the polymer during carbonization, thus the formation of a hollow cavity in CNP-2 to -7 samples. In contrast, the carbonization of SPC-1 with a thin silica layer leads to inward contraction and thus the formation of a solid core in CNP-1.

Understanding the time-dependent HSA process of composite particles opens the opportunity to further regulate the pore opening of CNP. Taking SPC-4 as an example, the intermediate structures were separated at reaction times of 5, 15, and 180 min. The surface composition of these SPCs was analyzed via XPS. As shown in fig. S17A, all the spectra show four typical peaks which represent Si 2p, C 1s, N 1s, and O 1s, indicating the existence of silica and polymer. Then, the ratio of Si/N on the surface of these composites was quantified as shown in fig. S17B. With increasing time, the ratio of Si/N is decreased from 2.0 (5 min) to 1.0 (15 min) and to 0.12 (180 min), indicating an A_I_-B (5 or 15 min) to A_I_-B-A_O_ assembly (180 min) transition. While the presence of the polymer outer shell in A_I_-B-A_O_ assembly led to the formation of CNP-4 with a rough surface and closed pores (Fig. 5B_4_), the carbonization of A_I_-B assembly in the absence of the polymer outer shell resulted in carbon nanoparticles with open pores (more obvious at 5 min; fig. S17, C_1_, D_1_, and E_1_).

### Understanding the impact of EDA amount on overall particle size change

By comparing the impact of EDA amount on the sizes of PC-*x* (fig. S7, black curve) and SPC-*x* (Fig. 3A, black curve), it can be seen that the trend of size change in the first six samples (*x* = 1 to 6) is similar. However, the particle size sharply increases in the other two SPC-*x* (*x* = 7 and 8) samples, different from corresponding PC-*x* samples in which the size of PC-7 is the smallest in all PC-*x* (SPC-8 has no obvious inner polymer core). The size change of PC-*x* (*x* = 1 to 8) affected by EDA has been explained before. The sharply increased sizes of SPC-7 and -8 could be attributed to the increased polymer content (Fig. 7B); thus, the increased size in the silica-polymer layer and outer shell overtakes the size reduction of the inner polymer core. CNP-*x* and SPC-*x* follow a similar trend of size change compared to SPC-*x*, except that the sizes are reduced because of contraction or calcination. Because of the removal of the outer polymer shell (A_O_) in A_I_-B-A_O_ during the calcination process, which is most significant in SPC-1 and SPC-8, the size difference is also the largest between CNP-1 and SPC-1, as well as CNP-8 and SPC-8.

### The implication of the HSA approach

Compared to literature reports on surfactant-free silica-polymer assembly (*18*, *20*, *22*, *23*, *25*, *26*, *59*, *60*), the major difference of the proposed HAS mechanism in this study is looking at the assembly at two length scales. One is the higher level of A_I_-B-A_O_ core-shell–type assembly, the other is a secondary level of silica-polymer assembly in the middle layer whose structure is correlated to the silica/polymer ratio. Understanding the HAS mechanism has important implications. First, the surface nanotopography of silica nanoparticles can be adjusted from spherical, ellipsoidal, 1D rodlike subunits to disordered pore structures, mainly determined by the EDA amount and silica/polymer ratio. Second, the aspect ratio of the 1D rodlike subunit can be controlled via the heterogeneous nucleation density of silica, and the pore size can also be regulated. Third, the hierarchical structures of carbon nanoparticles can be regulated by the A_I_-B-A_O_ spatial assembly, including the inner cavity, different shapes of inner pore channels, surface roughness, and pore openings (fig. S17).

The HSA strategy is simple, versatile, and reproducible. Taking SNP-4 as an example, the silica nanoparticles prepared in three batches exhibited similar structures (fig. S18). Their cavity and particles have no significant difference, indicating high reproducibility of the synthesis method. Besides, understanding the HSA mechanism allows fine control over the core-shell structures. For example, by adjusting the polymer inner core (A_I_) size by varying the APF polymerization time before the addition of the silica source, the hollow inner cavity size of silica nanoparticles can be enlarged with increasing A_I_ formation time (fig. S19). Moreover, the concentration of ammonia hydroxide used in the synthesis is another parameter that can adjust the structure of silica-polymer assembly. An increase in the overall solution alkalinity leads to faster nucleation of APF core with higher numbers and smaller sizes, thus both the particle and cavity sizes are reduced in the resultant silica nanoparticles (fig. S20).

**Fig. 9.**
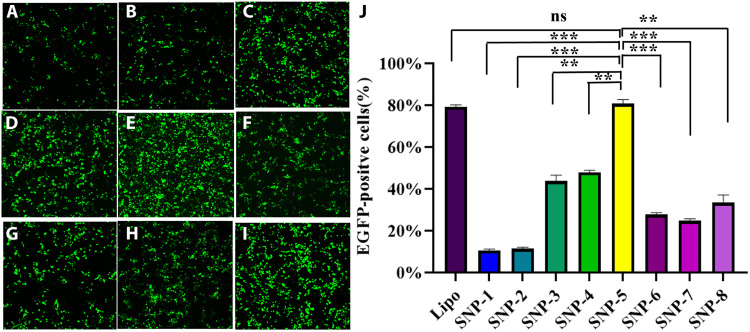
Plasmid DNA transfection evaluation. Confocal images (**A** to **I**) of EGFP gene expression mediated by SNP-1 to *8* and Lipofectamine 2000 (Lipo). (**J**) pDNA-EGFP transfection efficacy assay determined by flow cytometry. The bars are shown as means ± SD (*n* = 3), statistical analysis conducted by *t* tests with *P* > 0.05 showing no significant (ns) difference; *P < 0.05, **P < 0.01, and ***P < 0.001.

Precise control of surface topography especially in the nanoscale range is one of the important factors to improve the functionality both in biological activity and catalysis (*61*–*63*). As a proof of concept, we studied the effect of controllable nanotopography in silica nanoparticles on DNA transfection performance. The transfection efficiency of plasmid DNA encoding enhanced green fluorescent protein (pDNA-EGFP) was evaluated from the green fluorescence intensity of transfected GFP in human embryonic kidney (HEK) 293 T cells via confocal microscopy and flow cytometry. As shown in Fig. 9, SNP-5 exhibited the highest DNA transfection efficacy among the eight SNP-*x* samples, comparable to a commercial transfection agent Lipofectamine 2000. SNP-3, -4, -5, and -6 with rodlike surface nanotopography showed comparable DNA adsorption ability higher than SNP-1, -2, -7, and -8 (fig. S21A). Moreover, SNP-5 exhibited the lowest DNA release percentage compared with other SNP-*x* samples (fig. S21, B and C). In addition, the cellular uptake of SNP-4, -5, -6, -7, and -8 has no significant difference (fig. S22A) but is higher than SNP-1, -2, and -3 with low specific surface area (<100 m^2^/g; table S1). The above results suggest that SNP-5 with rodlike surface nanotopography is beneficial for DNA binding; its balanced specific surface area (337.7 m^2^/g), large pore size (14.4 nm), and particle size (173.7 nm) collectively contribute to the excellent DNA transfection performance.

One limitation of our work is although the aspect ratio of 1D rodlike subunits in silica nanoparticles can be regulated, the particle sizes are not kept constant. However, by understanding the HSA mechanism and the impact of EDA on the formation of each level in A_I_-B-A_O_ core-shell assembly, it is possible to adjust the inner polymer core size, such as changing the initial concentration of APF precursors, so that the interplay between the precursor concentration and EDA amount may lead to products with similar particle sizes. It is also possible to adjust the silica/polymer precursor ratio so that the silica subunit structure/particle size can be regulated. Another limitation is that the properties of the carbon nanoparticles with rough surfaces are not investigated. While more systematic studies are needed, this study has broadened the avenue toward the synthesis of versatile silica/carbon nanoparticles with more applications to explore in the future.

## MATERIALS AND METHODS

### Materials and bioreagents

3-AP (98%) and TEOS (reagent grade, 98%) were purchased from Sigma-Aldrich. Formaldehyde solution (ACS reagent, 37 wt %), ammonium hydroxide solution (ACS reagent, 28 to 30%), and ethylenediamine (GC, ≥99.5%) were purchased from Sigma-Aldrich. Anhydrous ethanol (>98%) was purchased from UQ Science Store. 3-(Trihydroxysilyl)propyl methylphosphonate (THPMP) and monosodium salt solution (50 wt % in H_2_O) were purchased from Sigma-Aldrich. Polyethylenimine (PEI), Branched, Mw 10,000 (Resin content, 99 wt %) was purchased from Polysciences Inc. HEK-293 T cells were purchased from American Type Culture Collection (CRL-3216). Dulbecco’s modified Eagle’s medium (DMEM) and fetal bovine serum (FBS; qualified, Australia) were purchased from Thermo Fisher Scientific. All chemicals and bioreagents were used as received without any further purification.

### Fabrication of silica-polymer composites

Colloidal SPCs were synthesized by the Stöber-like method in a surfactant-free system. Typically, 40 ml of ethanol, 10 ml of distilled water, 1.56 ml of ammonium hydroxide, and different amounts of ethylenediamine (*x* = 0.08 to 0.51 ml) were mixed together under vigorous stirring with a stirring rate of 300 to 350 rpm at 60°C. After that, 0.103 g of 3-AP and 1.2 ml of formaldehyde were added and stirred for 30 min to generate the APF core. Then, 1.165 ml of TEOS and 0.412 g of 3-AP were added to the abovementioned mixtures with stirring for 3 hours to generate SPCs. The final composites were obtained by centrifugation, ethanol washing, and vacuum drying at 45°C. The produced SPCs were named SPC-*x* (*x* = 1 to 9, corresponding to the EDA amount used in the synthesis of 0.08, 0.10, 0.12, 0.15, 0.22, 0.29, 0.31, and 0.51 ml, respectively.)

### Fabrication of silica nanoparticles

Silica nanoparticle was obtained via calcinating the above SPC-*x* in the air atmosphere at 550°C for 5 hours with a heating rate of 2°C/min. The products were named SNP-*x* (*x* = 1 to 9, corresponding to the EDA amount used in the synthesis of 0.08, 0.10, 0.12, 0.15, 0.22, 0.29, 0.31, and 0.51 ml, respectively.)

### Fabrication of carbon nanoparticles

Carbon nanoparticles were fabricated via carbonization of the above SPC-*x* in the nitrogen atmosphere at 700°C for 5 hours with a heating rate of 2°C/min. The silica was removed via 5% hydrofluoric acid etching. The products were named CNP-*x* (*x* = 1 to 9, corresponding to the EDA amount used in the synthesis of 0.08, 0.10, 0.12, 0.15, 0.22, 0.29, 0.31, and 0.51 ml, respectively.)

### PEI-modified silica nanoparticles functionalized with phosphonate group

Bare silica nanoparticles (SNP-3, -4, -5, -6, and -7) were first surface functionalized with THPMP, and then further modified with PEI similar to our previous methods (*21*, *64*).

For phosphonate functionalization, 30 mg of bare silica nanoparticles was dispersed into 10 ml of water via ultrasonication, and then mixed with 10 ml of 56 mM THPMP solution. The above mixture solution was stirred at 40°C for 2 hours. The solid products were collected by centrifugation, gently washed with water twice, and lastly dispersed in 5 ml of water for freeze-drying.

For PEI modification, 30 mg of the above freeze-dried phosphonate functionalized silica nanoparticles was redispersed into 10 ml of water via gently vortex and mixed with 10 ml of 50 mM carbonate buffer solution (pH 9.6) containing 30 mg of PEI (branched, 10 kDa). The mixture was stirred for 4 hours at room temperature, and the final solid product was obtained by centrifugation, gently washed with water, and redispersed into 5 ml of water for freeze-drying.

### Material characterization

TEM, field emission SEM, and HAADF-STEM with EDS detectors for elemental mapping analysis were taken on JEOL-7700, JEOL-7800, and HF-5000 microscopes, respectively. Samples for TEM and SEM analysis were prepared by dispersing samples in ethanol via ultrasonication and dried on a copper grid and silicon substrates, respectively. ET tilt series were carried out by tilting the specimen inside Tecnai F30 G2 TEM over an angular range of −60° to 60° under the electron beam and the images were processed via IMODE software. Nitrogen adsorption/desorption measurement was conducted using a Micromeritics TriStar II system at 77 K. Before the measurement, all samples were degassed for 12 hours under a vacuum environment at 170°C. The pore size and specific surface area of samples were calculated through the BJH and BET methods, respectively. The zeta potential of the polymer core in reactive solution and PEI-modified SNP-*x* dispersed in water was tested by DLS using a Zetasizer NanoZS from Malvern Instruments. XPS measurement was conducted using a Kratos Axis ULTRA.

### Plasmid DNA loading

Two microliters of pDNA-EGFP (500 ng/μl) was dispersed into 4 μl of phosphate-buffered saline (PBS). Then, 4 μl of PEI-modified SNP-*x* (*x* = 1 to 8, 5 mg/ml dispersed in PBS) was added into the above DNA solution with the weight ratio of DNA/SNP-*x* (1:20) and mixed via pipetting. The above mixtures were incubated at room temperature for 30 min, and then centrifuged at 15,000 rpm for 8 min. The supernatant was collected for the pDNA residual amount quantified via NanoDrop. The control sample is the mixture of 2 μl of pDNA dispersed in 8 μl of PBS. All samples were performed in triplicate.

### Gel electrophoresis

One microliter of pDNA (500 ng/μl) was mixed with PEI-modified SNP-*x* (*x* = 1 to 8, 5 mg/ml dispersed in PBS) at silica dosage of 0, 10, 20, and 40 μg with the total volume of 8 μl. The above mixture was incubated at room temperature for 30 min. Then, 2 μl of nucleic acid sample buffer (5×, Bio-Rad) was added into the mixture with a total volume of 10 μl to be loaded in each well.

Agarose gel is prepared as follows: 3.0 g of ultrapure agarose was added into 300 ml of Milli-Q water, and then boiled under microwave irradiation to be fully dissolved. Subsequently, 30 μl of SYBR Safe gel stain (10,000×) was added into the melted agarose solution via swirling the container. The cooled mixture was slowly poured into the gel box for solidification at room temperature. The solidified gel was transferred into the gel tank and fully covered with tris-acetate-EDTA buffer. Electrophoresis was carried out at 80 V for 50 min, and the bands were visualized on a UV tansilluminator (Bio-Rad). The intensity of DNA band in gel was further quantified via ImageJ software.

### Cellular uptake ability evaluation

The cellular uptake capability of PEI-modified SNP-*x* (*x* = 1 to 8) was evaluated in HEK-293 T cells and analyzed by inductively coupled plasma optical emission spectrometry (ICP-OES). Cells were seeded on the 12-well cell culture plate with a density of 1.4 × 10^5^ cells per well and cultured for 12 hours before adding SNP-*x* (*x* = 1 to 8). Two microliters of DNA (500 ng/μl) was first dispersed into 33 μl of PBS followed by the addition of 15 μl of PEI-modified SNP-*x* (*x* = 1 to 8, 4 mg/ml) to keep the total volume of 50 μl. The above mixture was dispersed into the medium with a final volume of 1 ml (the particle concentration is 60 μg/ml) and added to each well for 4-hour incubation. Afterward, cells were washed with warm PBS, treated with trypsin-EDTA (0.25%), isolated by centrifugation, and resuspended in PBS for cell density counting. After another centrifugation, cells were digested in 120 μl of distilled water via three cycles of freezing-thawing. The precipitates were collected via centrifugation, dried in a 50°C oven overnight, and dissolved in 150 μl of NaOH solution (1 M) at room temperature for 24 hours. Silicon content was detected via ICP-OES. All samples were performed in triplicate.

### In vitro plasmid DNA transfection experiment

HEK-293 T cells were seeded into a 12-well plate with a density of 1.2 × 10^5^ cells per well and cultured in DMEM containing 10% FBS for 24 hours before transfection. Two microliters of pDNA-EGFP (500 ng/μl) was dispersed into PBS with the addition of 15 μl of PEI-modified SNP-*x* (*x* = 1 to 8, 4 mg/ml) to keep the total volume of 50 μl. The above mixture was incubated at room temperature for 15 min, and then gently suspended into each well of the plate which contained 1 ml of DMEM with 10% FBS (final particle concentration is 60 μg/ml). After incubation at 37°C for 48 hours, the cells were directly imaged under confocal microscopy (Zeiss LSM 710) to assess the green fluorescent protein expression in cells, and the green fluorescence intensity was quantified by flow cytometry. Cells treated by a commercial transfection agent, Lipofectamine 2000 (Invitrogen), were used as a positive control, while cells without any treatment were used as a negative control. Experiments were performed in triplicate for each group.

## Supplementary Materials

### This PDF file includes:

Supplementary Text Figs. S1 to S22 Tables S1 and S2 References

## References

[1] G. M. Whitesides, B. Grzybowski, Self-assembly at all scales. Science 295, 2418–2421 (2002). 11923529 10.1126/science.1070821

[2] K. Schäfer, H. B. Kolli, M. K. Christensen, S. L. Bore, G. Diezemann, J. Gauss, G. Milano, R. Lund, M. Cascella, Supramolecular packing drives morphological transitions of charged surfactant micelles. Angew. Chem. Int. Ed. Engl. 59, 18591–18598 (2020). 32543728 10.1002/anie.202004522 PMC7589243

[3] D. Danino, Y. Talmon, H. Levy, G. Beinert, R. Zana, Branched threadlike micelles in an aqueous solution of a trimeric surfactant. Science 269, 1420–1421 (1995). 17731153 10.1126/science.269.5229.1420

[4] S. Jain, F. S. Bates, On the origins of morphological complexity in block copolymer surfactants. Science 300, 460–464 (2003). 12702869 10.1126/science.1082193

[5] S. A. Safran, P. Pincus, D. Andelman, Theory of spontaneous vesicle formation in surfactant mixtures. Science 248, 354–356 (1990). 17784490 10.1126/science.248.4953.354

[6] C. G. Goltner, M. Antonietti, Mesoporous materials by templating of liquid crystalline phases. Adv. Mater. 9, 431–436 (1997).

[7] T. Zhao, A. Elzatahry, X. Li, D. Zhao, Single-micelle-directed synthesis of mesoporous materials. Nat. Rev. Mater. 4, 775–791 (2019).

[8] S. Che, A. E. Garcia-Bennett, T. Yokoi, K. Sakamoto, H. Kunieda, O. Terasaki, T. Tatsumi, A novel anionic surfactant templating route for synthesizing mesoporous silica with unique structure. Nat. Mater. 2, 801–805 (2003). 14634644 10.1038/nmat1022

[9] Y. Wan, D. Zhao, On the controllable soft-templating approach to mesoporous silicates. Chem. Rev. 107, 2821–2860 (2007). 17580976 10.1021/cr068020s

[10] X. Zhang, Y. Li, C. Cao, Facile one-pot synthesis of mesoporous hierarchically structured silica/carbon nanomaterials. J. Mater. Chem. 22, 13918–13921 (2012).

[11] W. Stöber, A. Fink, E. Bohn, Controlled growth of monodisperse silica spheres in the micron size range. J. Colloid Interf. Sci. 26, 62–69 (1968).

[12] J. Liu, S. Z. Qiao, H. Liu, J. Chen, A. Orpe, D. Y. Zhao, G. Q. M. Lu, Extension of the Stöber method to the preparation of monodisperse resorcinol-formaldehyde resin polymer and carbon spheres. Angew. Chem. Int. Ed. Engl. 50, 5947–5951 (2011). 21630403 10.1002/anie.201102011

[13] A. H. Lu, G. P. Hao, Q. Sun, Can carbon spheres be created through the stöber method? Angew. Chem. Int. Ed. 50, 9023–9025 (2011). 10.1002/anie.201103514 21919134

[14] B. D. Credico, L. Vigano, C. Canevali, M. D’Arienzo, S. Mostoni, R. Nistico, R. Scotti, Silica nanoparticles self-assembly process in polymer composites: Towards advanced materials. Ceram. Int. 49, 26165–26181 (2023).

[15] J. N. Cha, G. D. Stucky, D. E. Morse, T. J. Deming, Biomimetic synthesis of ordered silica structures mediated by block copolypeptides. Nature 403, 289–292 (2000). 10659843 10.1038/35002038

[16] K. Nakanishi, N. Tanaka, Sol–gel with phase separation. Hierarchically porous materials optimized for high-performance liquid chromatography separations. Acc. Chem. Res. 40, 863–873 (2007). 17650924 10.1021/ar600034p

[17] O. Noonan, H. Zhang, H. Song, C. Xu, X. Huang, C. Yu, In situ stöber templating: Facile synthesis of hollow mesoporous carbon spheres from silica-polymer composites for ultra-high level in-cavity adsorption. J. Mater. Chem. A 4, 9063–9071 (2016).

[18] H. W. Zhang, O. Noonan, X. D. Huang, Y. N. Yang, C. Xu, L. Zhou, C. Z. Yu, Surfactant-free assembly of mesoporous carbon hollow spheres with large tunable pore sizes. ACS Nano 10, 4579–4586 (2016). 27050771 10.1021/acsnano.6b00723

[19] A. B. Fuertes, P. Valle-Vigon, M. Sevilla, One-step synthesis of silica@ resorcinol-formaldehyde spheres and their application for the fabrication of polymer and carbon capsules. Chem. Commun. 48, 6124–6126 (2012). 10.1039/c2cc32552g 22582187

[20] H. Song, Y. Nor, M. Yu, Y. Yang, J. Zhang, H. Zhang, C. Xu, N. Mitter, C. Yu, Silica nanopollens enhance adhesion for long-term bacterial inhibition. J. Am. Chem. Soc. 138, 6455–6462 (2016). 27139159 10.1021/jacs.6b00243

[21] H. Song, M. Yu, Y. Lu, Z. Gu, Y. Yang, M. Zhang, J. Y. Fu, C. Yu, Plasmid DNA delivery: Nanotopography matters. J. Am. Chem. Soc. 139, 18247–18254 (2017). 29151352 10.1021/jacs.7b08974

[22] J. Fu, Z. Gu, Y. Liu, J. Zhang, H. Song, Y. Yang, Y. Yang, O. Noonan, J. Tang, C. Yu, Bottom-up self-assembly of heterotrimeric nanoparticles and their secondary Janus generations. Chem. Sci. 10, 10388–10394 (2019). 32110328 10.1039/c9sc02961c PMC6988604

[23] Y. Liu, H. Zhang, O. Noonan, C. Xu, Y. Niu, Y. Yang, L. Zhou, X. Huang, C. Yu, Kinetically controlled assembly of nitrogen-doped invaginated carbon nanospheres with tunable mesopores. Chemistry 22, 14962–14967 (2016). 27593214 10.1002/chem.201602672

[24] H. Zhang, M. Yu, H. Song, O. Noonan, J. Zhang, Y. Yang, L. Zhou, C. Yu, Self-organized mesostructured hollow carbon nanoparticles via a surfactant-free sequential heterogeneous nucleation pathway. Chem. Mater. 27, 6297–6304 (2015).

[25] S.-C. Mei, G.-X. Huang, X.-H. Rui, L. Li, M.-K. Ke, X.-Q. Pan, Z.-H. Wang, X.-D. Yang, H.-Q. Yu, Y. Yu, Sequential assembly tailored interior of porous carbon spheres for boosted water decontamination through peroxymonosulfate activation. Adv. Funct. Mater. 32, 2111184 (2022).

[26] S. C. Mei, X. H. Rui, L. Li, G. X. Huang, X. Q. Pan, M. K. Ke, Z. H. Wang, H. Q. Yu, Y. Yu, Quantitative coassembly for precise synthesis of mesoporous nanospheres with pore structure-dependent catalytic performance. Adv. Mater. 33, e2103130 (2021). 34510574 10.1002/adma.202103130

[27] J. N. Israelachvili, D. J. Mitchell, B. W. Ninham, Theory of self-assembly of hydrocarbon amphiphiles into micelles and bilayers. J. Chem. Soc. Faraday Trans. 72, 1525–1568 (1976).

[28] G. S. Attard, J. C. Glyde, C. G. Goltner, Liquid-crystalline phases as templates for the synthesis of mesoporous silica. Nature 378, 366–368 (1995).

[29] C. T. Kresge, M. E. Leonowicz, W. J. Roth, J. C. Vartuli, J. S. Beck, Ordered mesoporous molecular-sieves synthesized by a liquid-crystal template mechanism. Nature 359, 710–712 (1992).

[30] W. Wang, P. Wang, X. Tang, A. A. Elzatahry, S. W. Wang, D. Al-Dahyan, M. Zhao, C. Yao, C.-T. Hung, X. Zhu, T. C. Zhao, X. Li, F. Zhang, D. Zhao, Facile synthesis of uniform virus-like mesoporous silica nanoparticles for enhanced cellular internalization. ACS Cent. Sci. 3, 839–846 (2017). 28852697 10.1021/acscentsci.7b00257 PMC5571464

[31] Z. Zhao, Y. Zhao, R. Lin, Y. Ma, L. Wang, L. Liu, K. Lan, J. Zhang, H. Chen, M. Liu, F. Bu, P. Zhang, L. Peng, X. Zhang, Y. Liu, C.-T. Hung, A. Dong, W. Li, D. Zhao, Modular super-assembly of hierarchical superstructures from monomicelle building blocks. Sci. Adv. 8, eabo0283 (2022). 35559684 10.1126/sciadv.abo0283 PMC9106296

[32] Z. Zhao, L. Duan, Y. Zhao, L. Wang, J. Zhang, F. Bu, Z. Sun, T. Zhang, M. Liu, H. Chen, Y. Yang, K. Lan, Z. Lv, L. Zu, P. Zhang, R. Che, Y. Tang, D. Chao, W. Li, D. Zhao, Constructing unique mesoporous carbon superstructures via monomicelle interface confined assembly. J. Am. Chem. Soc. 144, 11767–11777 (2022). 35731994 10.1021/jacs.2c03814

[33] H. Li, L. Chen, X. Li, D. Sun, H. Zhang, Recent progress on asymmetric carbon-and silica-based nanomaterials: From synthetic strategies to their applications. Nanomicro Lett. 14, 45 (2022). 35038075 10.1007/s40820-021-00789-y PMC8764017

[34] Y. Yang, M. Zhang, H. Song, C. Yu, Silica-based nanoparticles for biomedical applications: From nanocarriers to biomodulators. Acc. Chem. Res. 53, 1545–1556 (2020). 32667182 10.1021/acs.accounts.0c00280

[35] A. Saleem, Y. Z. Zhang, M. Usman, M. Haris, P. Li, Tailored architectures of mesoporous carbon nanostructures: From synthesis to applications. Nano Today 46, 101607 (2022).

[36] R. K. Kankala, Y. H. Han, J. Na, C. H. Lee, Z. Q. Sun, S. B. Wang, T. Kimura, Y. S. Ok, Y. Yamauchi, A. Z. Chen, K. C.-W. Wu, Nanoarchitectured structure and surface biofunctionality of mesoporous silica nanoparticles. Adv. Mater. 32, 1907035 (2020). 10.1002/adma.201907035 32319133

[37] J. W. F. To, Z. Chen, H. B. Yao, J. J. He, K. Kim, H. H. Chou, L. J. Pan, J. Wilcox, Y. Cui, Z. Bao, Ultrahigh surface area three-dimensional porous graphitic carbon from conjugated polymeric molecular framework. ACS Cent. Sci. 1, 68–76 (2015). 27162953 10.1021/acscentsci.5b00149 PMC4827563

[38] Y. Han, J. Y. Ying, Generalized fluorocarbon-surfactant-mediated synthesis of nanoparticles with various mesoporous structures. Angew. Chem. Int. Ed. 44, 288–292 (2005). 10.1002/anie.200460892 15614899

[39] M. Thommes, A. V. Neimark, J. P. Olivier, F. Rodriguez-Reinoso, J. Rouquerol, K. S. W. Sing, Physisorption of gases, with special reference to the evaluation of surface area and pore size distribution (IUPAC Technical Report). Pure Appl. Chem. 87, 1051–1069 (2015).

[40] P. Van der Voort, P. I. Ravikovitch, K. P. De Jong, A. V. Neimark, A. H. Janssen, M. Benjelloun, E. Van Bavel, P. Cool, B. M. Weckhuysen, E. F. Vansant, Plugged hexagonal templated silica: A unique micro- and mesoporous composite material with internal silica nanocapsules. Chem. Commun. (Camb) 9, 1010–1011 (2002). 10.1039/b201424f 12123048

[41] H. Song, Y. Yang, J. Geng, Z. Gu, J. Zou, C. Yu, Electron tomography: A unique tool solving intricate hollow nanostructures. Adv. Mater. 31, 1801564 (2019). 10.1002/adma.201801564 30160340

[42] R. Leary, P. A. Midgley, J. M. Thomas, Recent advances in the application of electron tomography to materials chemistry. Acc. Chem. Res. 45, 1782–1791 (2012). 22897395 10.1021/ar3001102

[43] D. J. De Rosier, A. Klug, Reconstruction of three dimensional structures from electron micrographs. Nature 217, 130–134 (1968). 23610788 10.1038/217130a0

[44] S. L. J. Thomä, S. W. Krauss, M. Eckardt, P. Chater, M. Zobel, Atomic insight into hydration shells around facetted nanoparticles. Nat. Commun. 10, 995 (2019). 30824693 10.1038/s41467-019-09007-1 PMC6397290

[45] N. P. Wickramaratne, J. T. Xu, M. Wang, L. Zhu, L. M. Dai, M. Jaroniec, Nitrogen enriched porous carbon spheres: Attractive materials for supercapacitor electrodes and CO_2_ adsorption. Chem. Mater. 26, 2820–2828 (2014).

[46] X. H. Cui, S. Mao, M. Liu, H. Yuan, Y. Du, Mechanism of surfactant micelle formation. Langmuir 24, 10771–10775 (2008). 18729337 10.1021/la801705y

[47] S. Ghosh, A. Ray, N. Pramanik, Self-assembly of surfactants: An overview on general aspects of amphiphiles. Biophys. Chem. 265, 106429 (2020). 32693319 10.1016/j.bpc.2020.106429

[48] C. C. M. C. Carcouët, M. W. P. van de Put, B. Mezari, P. C. M. M. Magusin, J. Laven, P. H. H. Bomans, H. Friedrich, A. C. C. Esteves, N. A. J. M. Sommerdijk, R. A. T. M. van Benthem, G. de With, Nucleation and growth of monodisperse silica nanoparticles. Nano Lett. 14, 1433–1438 (2014). 24499132 10.1021/nl404550d

[49] Y. Han, Z. Lu, Z. Teng, J. Liang, Z. Guo, D. Wang, M.-Y. Han, W. Yang, Unraveling the growth mechanism of silica particles in the stöber method: In situ seeded growth model. Langmuir 33, 5879–5890 (2017). 28514596 10.1021/acs.langmuir.7b01140

[50] D. L. Green, S. Jayasundara, Y. F. Lam, M. T. Harris, Chemical reaction kinetics leading to the first Stober silica nanoparticles – NMR and SAXS investigation. J. Non Cryst. Solids 315, 166–179 (2003).

[51] T. Dwars, E. Paetzold, G. Oehme, Reactions in micellar systems. Angew. Chem. Int. Ed. Engl. 44, 7174–7199 (2005). 16276555 10.1002/anie.200501365

[52] A. Sorrenti, O. Illa, R. M. Ortuno, Amphiphiles in aqueous solution: Well beyond a soap bubble. Chem. Soc. Rev. 42, 8200–8219 (2013). 23884241 10.1039/c3cs60151j

[53] A. E. C. Palmqvist, Synthesis of ordered mesoporous materials using surfactant liquid crystals or micellar solutions. Curr. Opin. Colloid Interface Sci. 8, 145–155 (2003).

[54] C.-F. Cheng, Z. Luan, J. Klinowski, The role of surfactant micelles in the synthesis of the mesoporous molecular-sieve MCM-41. Langmuir 11, 2815–2819 (1995).

[55] P. Innocenzi, L. Malfatti, T. Kldchob, P. Falcaro, Order-disorder in self-assembled mesostructured silica films: A concepts review. Chem. Mater. 21, 2555–2564 (2009).

[56] C. Xu, C. Lei, Y. Wang, C. Yu, Dendritic mesoporous nanoparticles: Structure, synthesis and properties. Angew. Chem. Int. Ed. Engl. 61, e202112752 (2022). 34837444 10.1002/anie.202112752

[57] Y. Wang, B. Zhang, X. Ding, X. Du, Dendritic mesoporous organosilica nanoparticles (DMONs): Chemical composition, structural architecture, and promising applications. Nano Today 39, 101231 (2021).

[58] K. L. Cao, A. F. Arif, K. Kamikubo, T. Izawa, H. Iwasaki, T. Ogi, Controllable synthesis of carbon-coated SiO*_x_* particles through a simultaneous reaction between the hydrolysis-condensation of tetramethyl orthosilicate and the polymerization of 3-aminophenol. Langmuir 35, 13681–13692 (2019). 31558027 10.1021/acs.langmuir.9b02599

[59] J. Fu, J. Jiao, H. Song, Z. Gu, Y. Liu, J. Geng, K. S. Jack, A. Du, J. Tang, C. Yu, Fractal-in-a-sphere: Confined self-assembly of fractal silica nanoparticles. Chem. Mater. 32, 341–347 (2020).

[60] L. Xie, M. Yan, T. Liu, K. Gong, X. Luo, B. Qiu, J. Zeng, Q. Liang, S. Zhou, Y. He, W. Zhang, Y. Jiang, Y. Yu, J. Tang, K. Liang, D. Zhao, B. Kong, Kinetics-controlled super-assembly of asymmetric porous and hollow carbon nanoparticles as light-sensitive smart nanovehicles. J. Am. Chem. Soc. 144, 1634–1646 (2022). 35014789 10.1021/jacs.1c10391

[61] M. Gulumian, C. Andraos, A. Afantitis, T. Puzyn, N. J. Coville, Importance of surface topography in both biological activity and catalysis of nanomaterials: Can catalysis by design guide safe by design? Int. J. Mol. Sci. 22, 8347 (2021). 34361117 10.3390/ijms22158347 PMC8348784

[62] R. K. Singh, J. C. Knowles, H.-W. Kim, Advances in nanoparticle development for improved therapeutics delivery: Nanoscale topographical aspect. J. Tissue. Eng. 10, 2041731419877528 (2019). 31555432 10.1177/2041731419877528 PMC6749784

[63] J. A. Finbloom, C. Huynh, X. Huang, T. A. Desai, Bioinspired nanotopographical design of drug delivery systems. Nat. Rev. Bioeng. 1, 139–152 (2023).

[64] D. Cheng, S. Theivendran, J. Tang, L. Cai, J. Zhang, H. Song, C. Yu, Surface chemistry of spiky silica nanoparticles tailors polyethyleneimine binding and intracellular DNA delivery. J. Colloid Interf. Sci. 628, 297–305 (2022). 10.1016/j.jcis.2022.08.038 35998455

[65] J. Zhao, W. Niu, L. Zhang, H. Cai, M. Han, Y. Yuan, S. Majeed, S. Anjum, G. Xu, A template-free and surfactant-free method for high-yield synthesis of highly monodisperse 3-aminophenol-formaldehyde resin and carbon nano/microspheres. Macromolecules 46, 140–145 (2013).

