# Supplementary material for: A hierarchical spatial assembly approach of silica-polymer composites leads to versatile silica/carbon nanoparticles
Source: Sci Adv. 2023 Oct 4;9(40):eadi7502. doi: 10.1126/sciadv.adi7502 (PMC10550229; doi:10.1126/sciadv.adi7502)
Supplement: Supplementary file 1 — Supplementary Text Figs. S1 to S22 Tables S1 and S2 References [file sciadv.adi7502_sm.pdf]

Science Advances
AAAS

Supplementary Materials for

A hierarchical spatial assembly approach of silica-polymer composites leads to versatile silica/carbon nanoparticles

Dan Cheng *et al.*

Corresponding author: Chenzhong Yu, c.yu@uq.edu.au, Hao Song, h.song6@uq.edu.au

Sci. Adv. **9**, eadi7502 (2023)
DOI: 10.1126/sciadv.adi7502

This PDF file includes:

Supplementary Text
Figs. S1 to S22
Tables S1 and S2
References

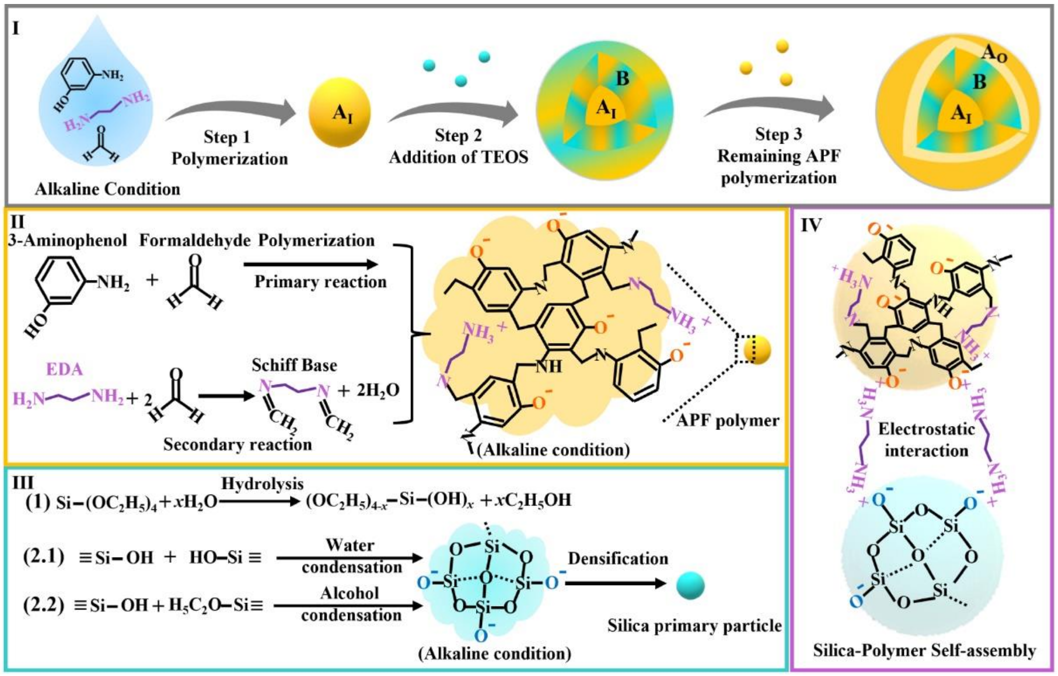

Fig. S1. Synthesis process of A_I-B-A_O core-shell type of silica-polymer composite. Three steps (I) is included: In step 1, the polymerization of 3-aminophenol and formaldehyde in the presence of EDA leads to the formation of polymer inner core (A_I) (*65*) with a Schiff base doped APF polymer network under alkaline condition (*23*) (II). In step 2, the hydrolysis and condensation of added TEOS precursors generate silica primary particles (*48-50*) (III), which triggers the formation of the middle layer (B) by silica-polymer self-assembly assisted by the EDA via electrostatic interaction (IV). In step 3, the polymer outer shell (A_O) is formed by the remaining APF polymer.

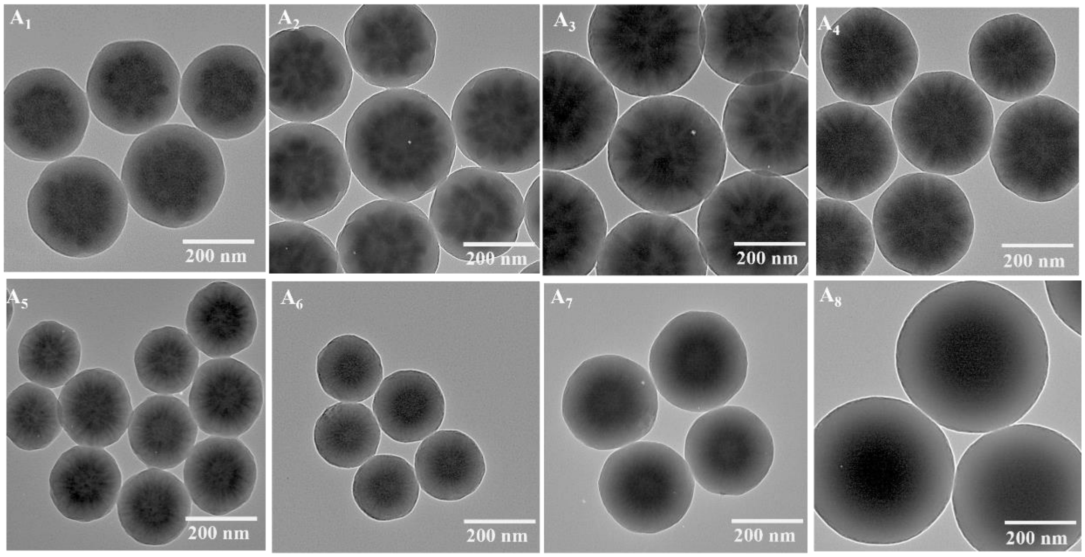

Fig. S2. TEM images (A_1) ~ (A_8) of SPC-*x* (x=*1~8*)

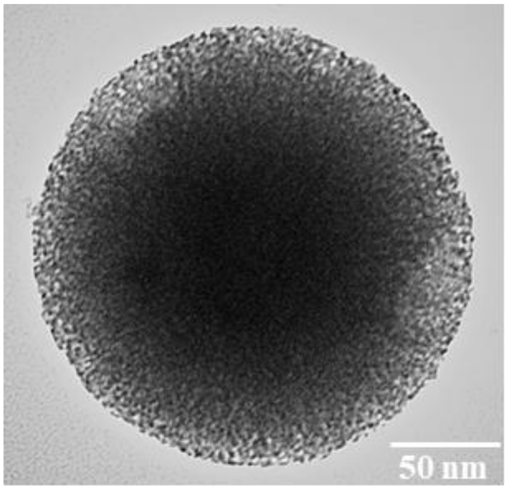

Fig. S3. TEM image of SNP-*8* in high magnification

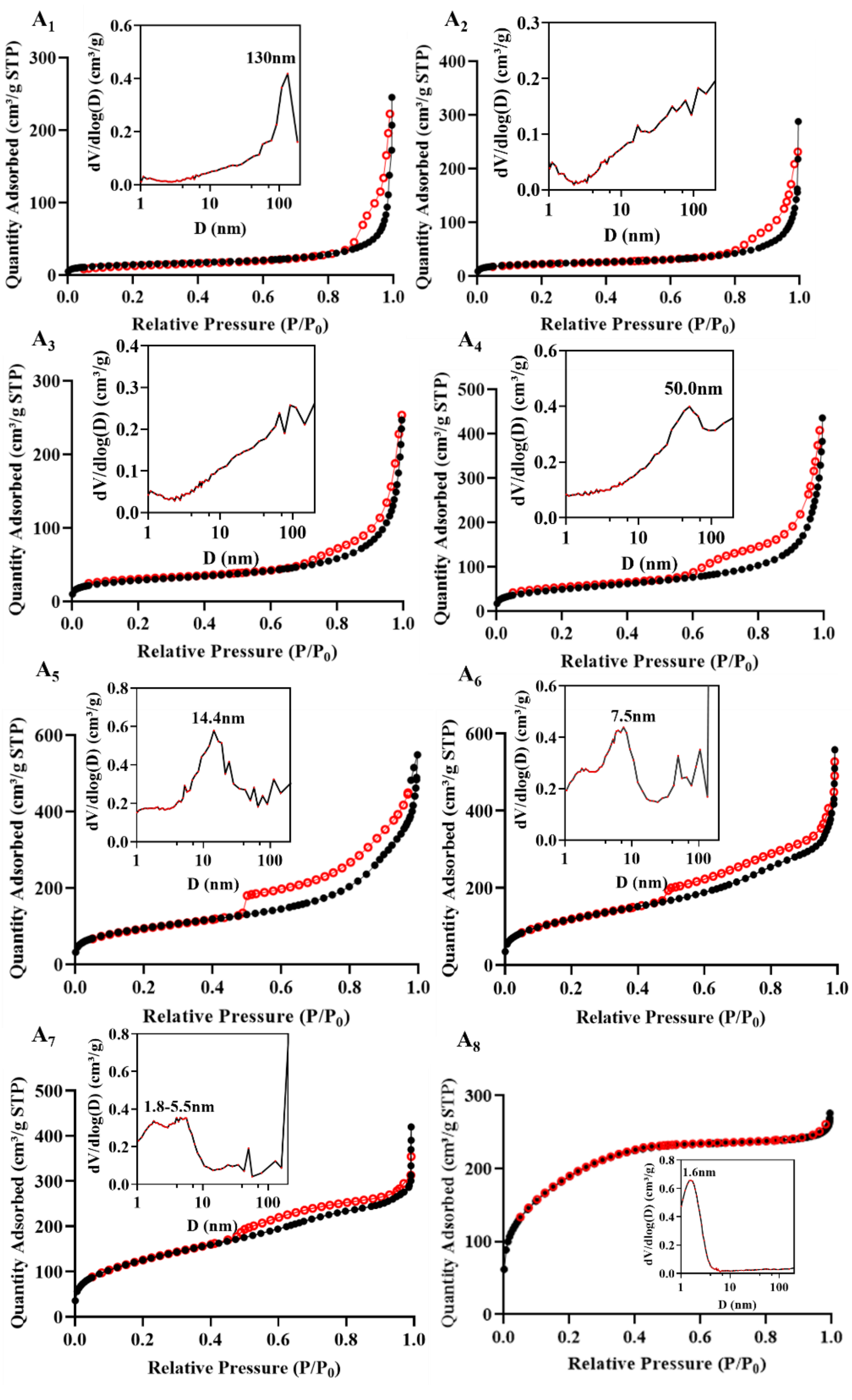

Fig. S4. Textual properties of silica nanoparticles: Nitrogen adsorption/desorption isotherms and the corresponding pore size distributions (inset) of SNP-*x* (x=1~8, A_1-A_8)

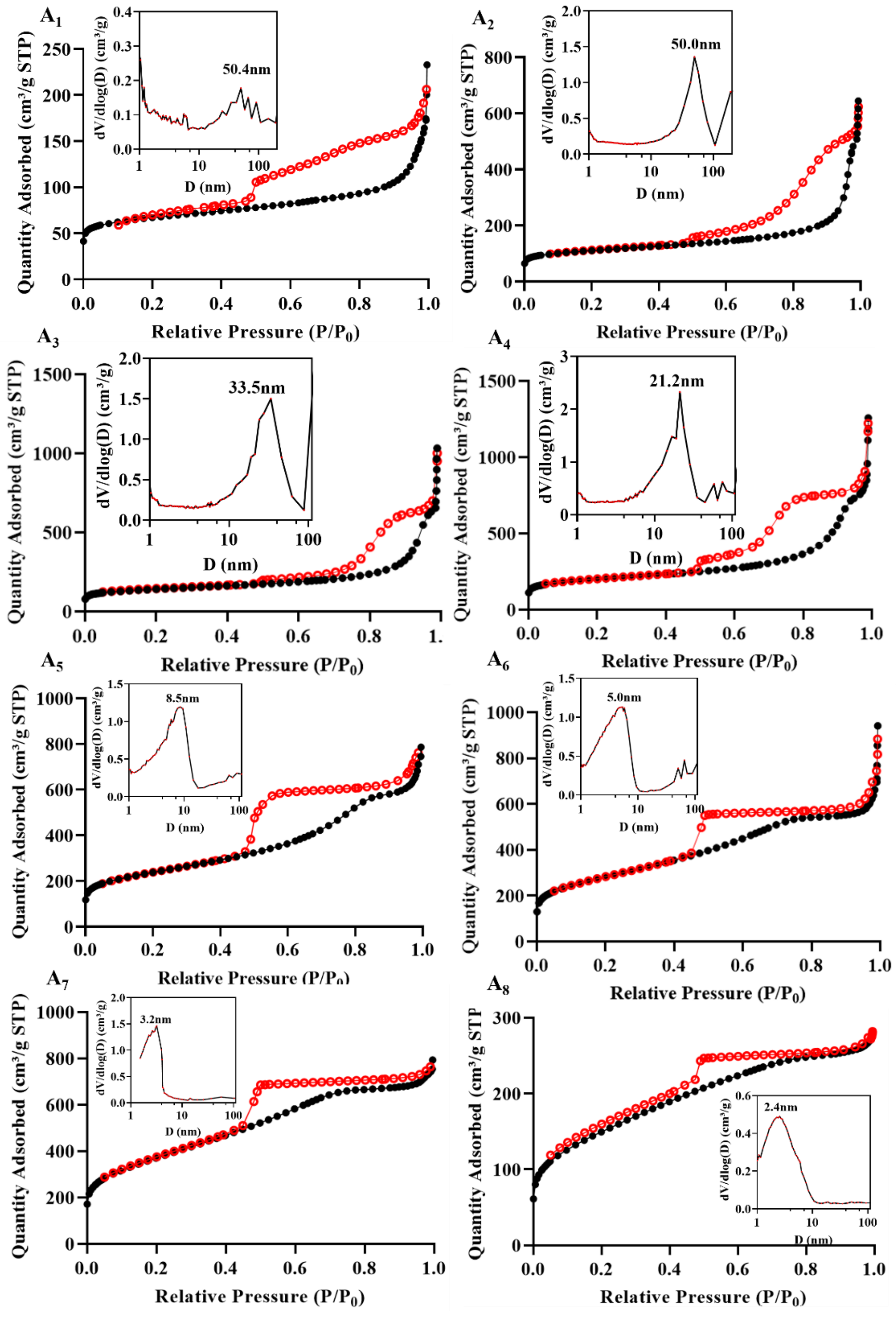

Fig. S5. Textual properties of carbon nanoparticles: Nitrogen adsorption/desorption isotherms and the corresponding pore size distributions (inset) of CNP-*x* (*x=1~8*, A_1-A_8)

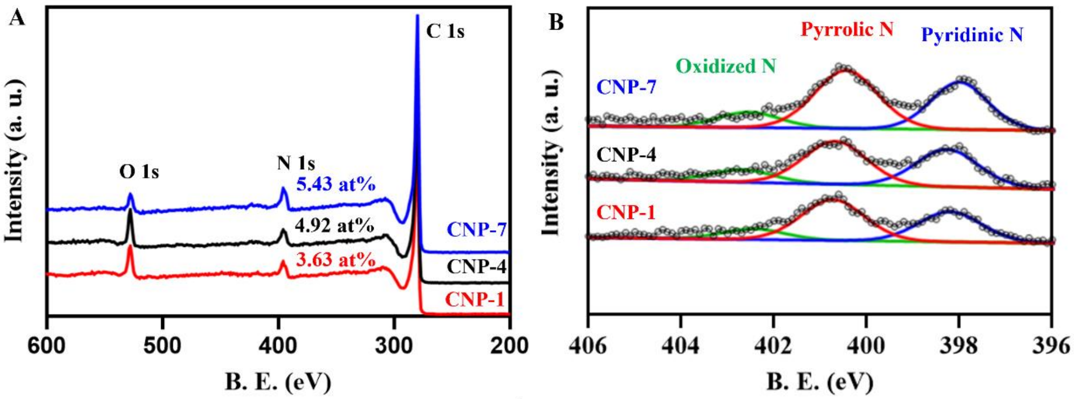

Fig. S6. XPS analysis. (A) The wide XPS survey spectrum, (B) N 1s spectrum of CNP-*1,4,7*.

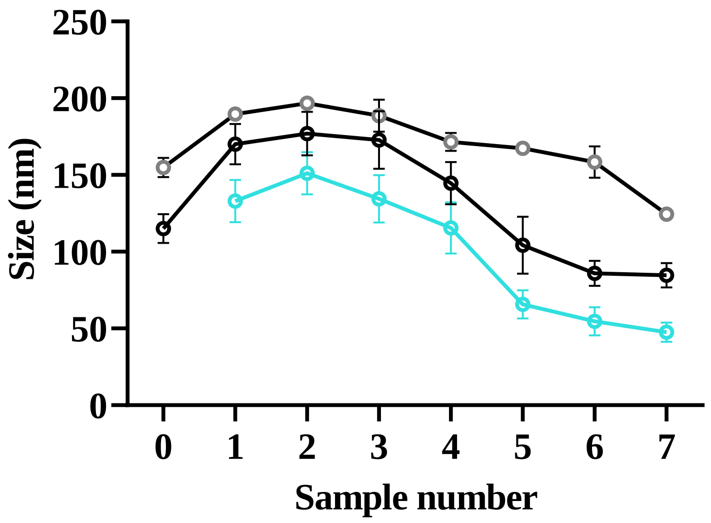

Fig. S7. Analysis of particle and hollow cavity sizes. The particle sizes of PC-*x* (x=*0~7*, 0 is the sample without adding EDA) measured from TEM images (black curve) and via DLS (grey curve). From TEM, the size of PC-*0* is 115.2 nm. After adding EDA, the size of PC firstly increases to 170.0 and 176.9 nm (PC-*1*, PC-*2*), then decrease continuously from 172.8 nm (PC-*3*) into 84.6 nm (PC-*7*). The size trend of PC measured from DLS is similar to the one of TEM, as shown in grey line, the size of PC-*0* is 154.9 nm. After adding EDA, the size firstly increases to 189.6 and 196.7 nm (PC-*1*, PC-*2*), then decrease continuously from 188.7 nm (PC-*3*) into 124.4 nm (PC-*7*). Hollow inner cavity sizes of SNP-*x* measured from TEM images in Fig. 4 are shown in blue curve.

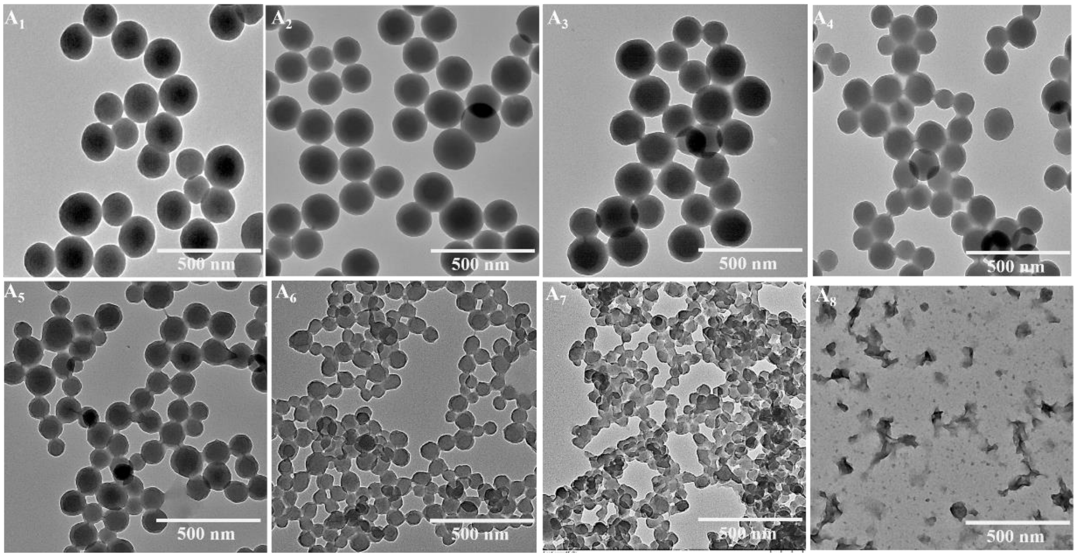

Fig. S8. TEM images (A_1) - (A_8) of PC-*x* (*x=1~8*).

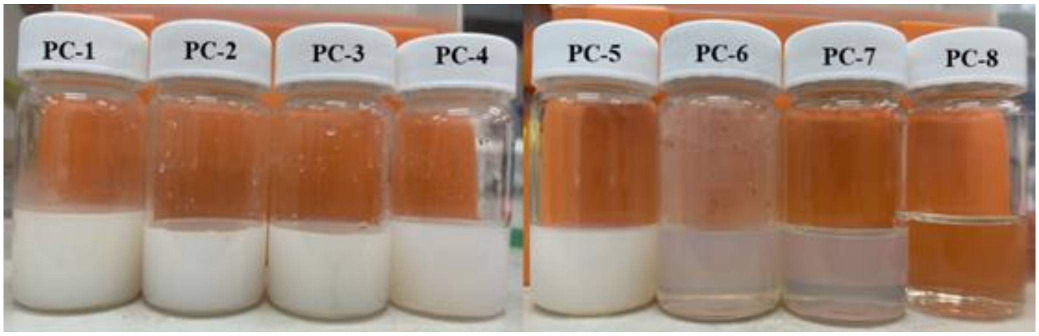

Fig. S9. Images of the synthesis solution of PC-*x* (*x=1~8*). Samples were obtained at the reactive time of 30 min, before adding silica precursor. The milky dispersion of PC-*1*~5 is in accordance with the formation of colloidal APF nanoparticles as shown in Fig. S8. The solution became less cloudy in PC-*6* and -*7*, and nearly transparent in PC-*8*, indicating the gradually retarded polymerization of APF polymer.

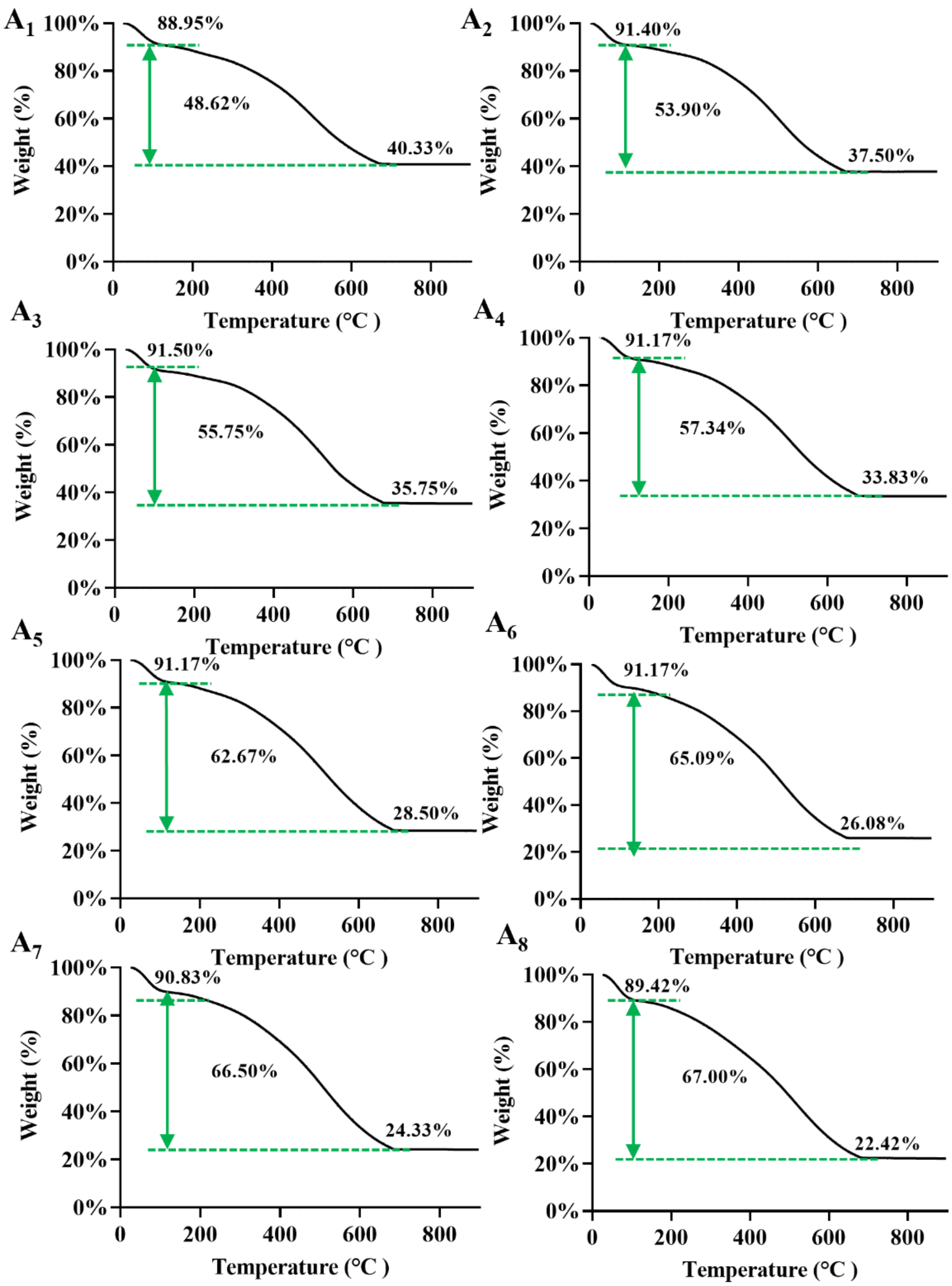

Fig. S10. TGA profiles of SPC-*x* (x=*1~8*, A_1-A_8). The weight loss before 100 °C in the first region is attributed to physical adsorbed water. The second region from 100 to 700 °C is mainly attributed to the thermal decomposition of polymer and condensation of silica. The weight loss after 700 °C is negligible, due to stable silica structure.

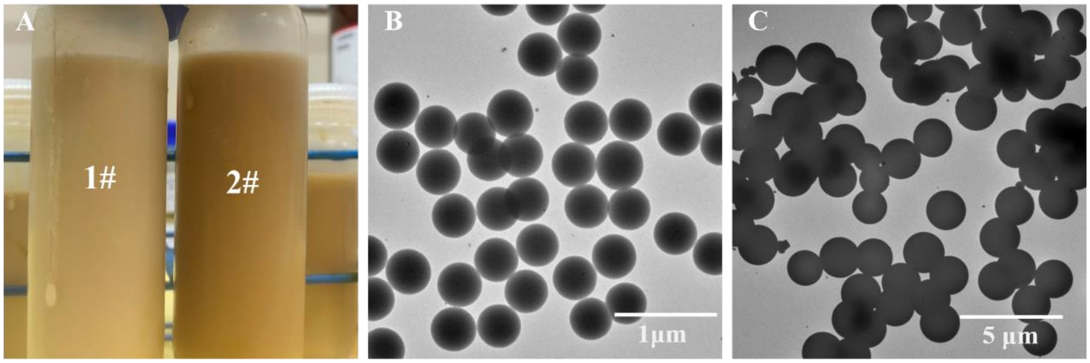

Fig. S11. The impact of TEOS on the growth of SPC-*8*. (A) Photos of the reactive solutions of SPC-*8* with (1#) and without (2#) adding TEOS in the second step. TEM images of SPC-*8* with (B) and without (C) adding TEOS in the second step.

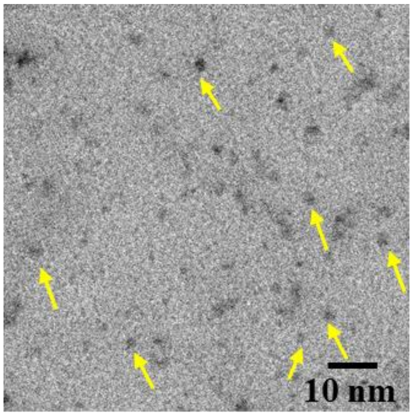

Fig. S12. Cryo-TEM image of silica primary particles.

Near-spherical particles with a mean size of 2.41± 0.47 nm were observed when only TEOS was added into the reactive solution after 15 minutes, indicating the existence of SPP. The particle size was measured via Image J software based on the TEM image.

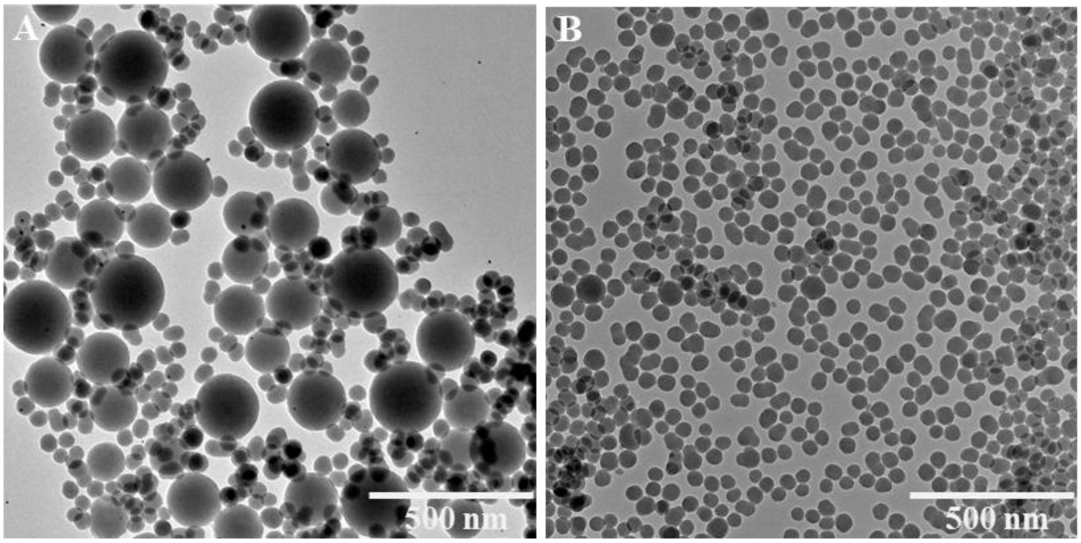

Fig. S13. The role of EDA in the HSA mechanism. TEM images of separated silica and polymer spheres without using EDA (A) and silica nanoparticle after calcination to remove the polymer (B). The average size of polymer in (A) is ~150 nm while that of silica in both (A) and (B) is ~ 50 nm.

The polymer can be fully decomposed after calcination in air, while silica is thermally stable. The absence of the nanoparticles with a larger size (~150 nm) after calcination indicates that they are polymers rather than silica.

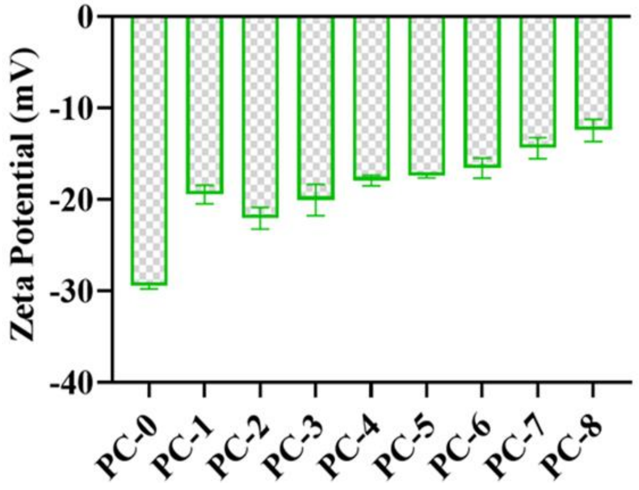

Fig. S14. Zeta potential of APF spheres. Without EDA: PC-*0*; with adding different amount of EDA: PC-*1~8*. Measured in water.

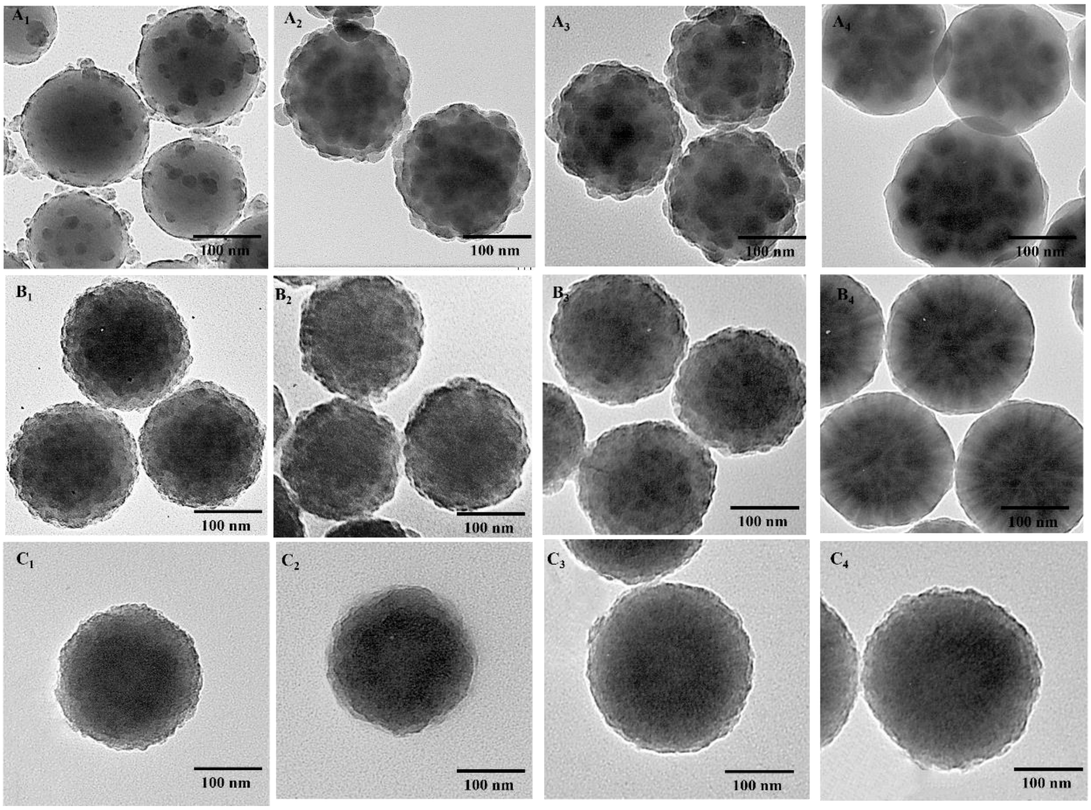

Fig. S15. Time-dependent structural analysis of SPC-*1, 4, 7*. TEM images of SPC-*1* (A_1-A_4), SPC-*4* (B_1-B_4) and SPC-*7* (C_1-C_4) obtained at 2 min (A_1, B_1, C_1), 5 min (A_2, B_2, C_2), 15 min (A_3, B_3, C_3) and 30 min (A_4, B_4, C_4) after adding silica precursor.

In bright-field TEM images, usually silica shows darker contrast than polymer. Randomly distributed silica islands with spherical morphology were observed on the surface of preformed APF core in SPC-*1* at 2 min (Fig. S15A_1), while the density of silica islands increased at 5 min (Fig. S15A_2). Later, APF polymer was filled into the interspaces between these silica islands which was more obvious at 30 min (Fig. S15A_4) where the rough silica/polymer nanosphere derived from exposed silica turned into smooth nanosphere. At the same reaction time, a higher density of silica islands with relatively smaller sizes were observed in SPC-*4* (Fig. S15, B_1, B_2) than SPC-*1*. Besides, the APF polymer co-assembled with silica at early stage (Fig. S15, B_1-B_3) which could be observed from the relative smooth outer surface of silica-polymer composite nanosphere compared to SPC-*1*. The surface roughness observed in SPC-*1* and SPC-*4* was not obvious in SPC-*7* (Fig. S15, C_1-C_3) indicating the highly polymer rich outer surface of SPC-*7* and the much earlier co-assembling behavior of silica and polymer compared to SPC-*1* and SPC-*4*.

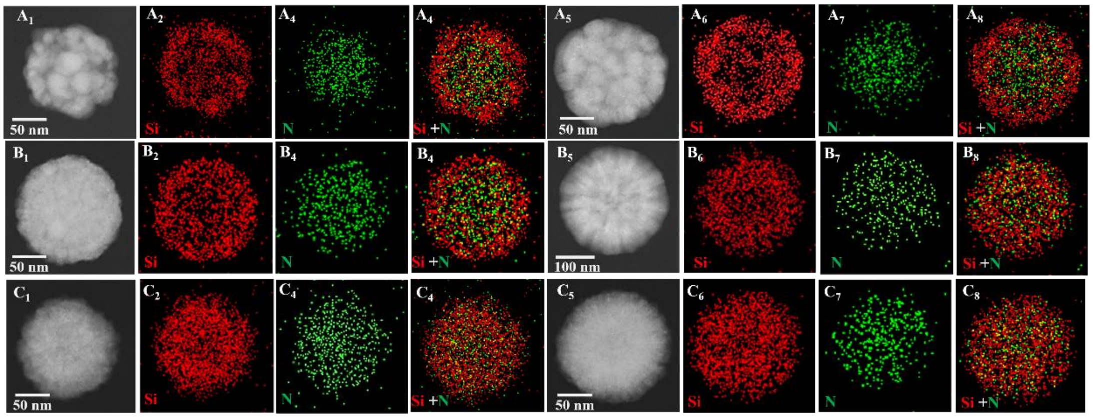

Fig. S16. Time-dependent compositional analysis of SPC-*1, 4, 7*. HAADF-STEM images (A_1-C_1 and A_5-C_5), elemental mapping images of silica (A_2-C_2 and A_6-C_6), polymer (A_4-C_4 and A_7-C_7) and the merged images of silica and polymer (A_4-C_4 and A_8-C_8) in SPC-*1,4,7* at 5 and 15 min. The distribution of silica is represented by the silicon element with red color, the distribution of APF polymer is represented by the nitrogen element with green color which is derived from amine groups in APF polymer.

To differentiate the distribution of polymer and silica in the composite, HAADF-STEM and elemental mapping images were recorded for intermediate SPC-*1,4,7* obtained at 5 and 15 min. For SPC-*1* obtained at 5 min, the spherical silica islands with brighter contrast were observed in HAADF-STEM image (Fig. S16A_1). The corresponding elemental mapping images (Si, N and Si+N, Fig. S16, A_2, A_3 and A_4, respectively) shows that the polymer core is surrounded by spherical nanodomains, consistent with the findings in Fig. S15. The silica rich outer shell is also seen for SPC-*1* obtained at 15 min (Fig. S16, A_5-A_8). For SPC-*4* (Fig. S16, B_1-B_8), the size of silica nanodomain is smaller while the density is higher compared with SPC-*1*. Moreover, the radially aligned silica nanodomain is more obvious at 15 min (Fig. S16B_5) than that at 5 min (Fig. S16B_1). In addition, the overlap between silica and polymer is more even than SPC-*1*, indicative of simultaneous silica-polymer assembly. For SPC-*7*, the silica nanodomain size is the smallest among three SPC samples under study. The silica and polymer distribution are more homogeneous, and the aligned growth of silica is not evident (Fig. S16, B_1-B_8**)**.

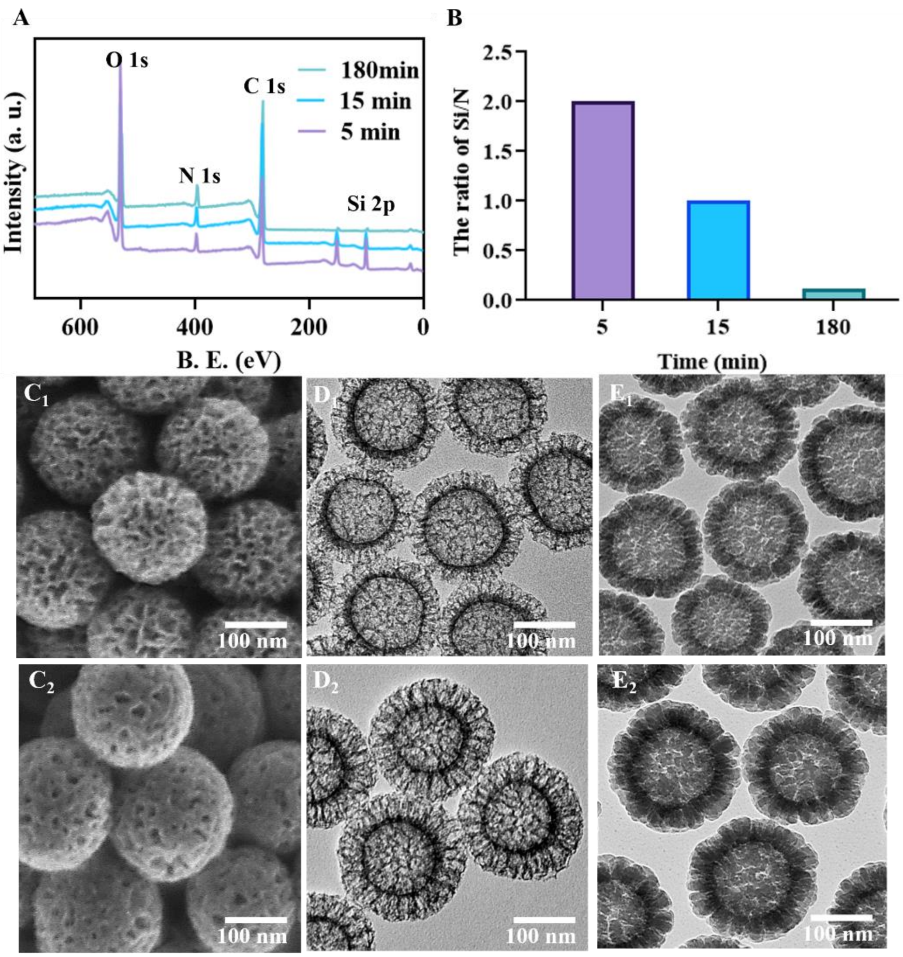

Fig. S17. Control over the pore openings of carbon nanoparticles. (A) The XPS survey spectra and (B) corresponding Si/N ratio of the intermediate SPC-*4* obtained at the reaction time of 5, 15 and 180 min. (C_1, C_2) SEM and (D_1, D_2) TEM images of carbon nanoparticles, and (E_1, E_2) TEM images of silica nanoparticles derived from intermediate SPC-*4* obtained at 5 and 15 min, respectively.

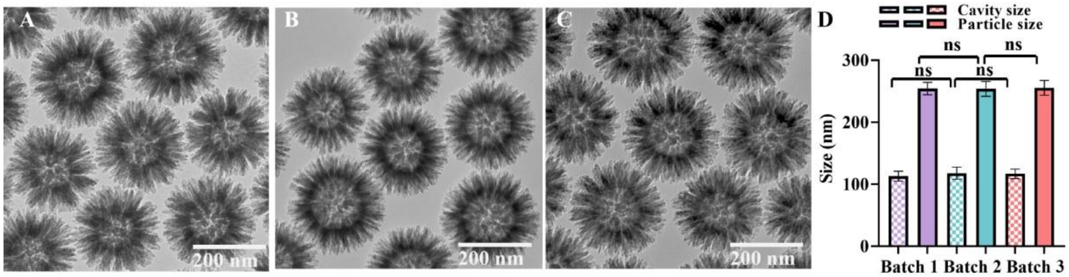

Fig. S18. The reproducibility of the synthesis method. TEM images of SNP-*4* obtained in three different batches (A~C). The cavity and particle sizes (D) measured from ~50 particles in TEM images via Image J software. The bars are shown as mean ± SD, statistical analysis conducted by t-tests with $p > 0.05$ showing no significant difference (ns).

From TEM images (Fig. S18, A~C), silica nanoparticles exhibit similar structures. The cavity and particle sizes of silica nanoparticles (Fig. S18D) prepared in three batches have no significant difference ($p > 0.05$). Cavity and particles are 113.1 ± 8.1 and 254.4 ± 10.0 nm in Batch 1, 117.7 ± 9.8 and 253.8± 12.0 nm in Batch 2, 116.8 ± 7.7 and 255.6 ± 11.9 nm in Batch 3.

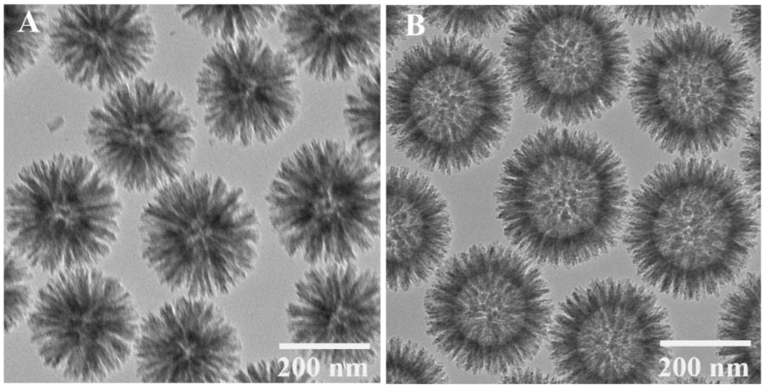

Fig. S19. The impact of polymer core formation time on the structure of silica nanoparticles. TEM images of SNP-*4* synthesized at the APF core formation time of 15 (A) and 50 min (B).

The hollow inner cavity size of silica nanoparticles was measured to be ~80 and ~ 150 nm when the APF polymerization time was 15 and 50 min, respectively. The average particle size was ~250 and ~275 nm, respectively. By comparing the cavity and particle size of SNP-*4* (Fig. 4A_4) synthesized at the APF core formation time of 30 min (115 and 254 nm, respectively, Fig. S7 and Fig. 3A), it is concluded that the size of APF core increases with the polymerization time, leading to enlarged cavity size in the silica nanoparticles and reduced spiky length due to over consumption of APF in the inner core.

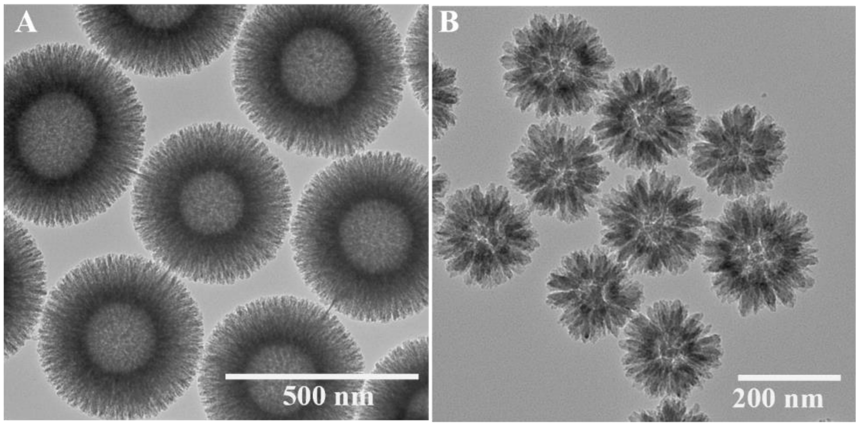

Fig. S20. The impact of ammonia amount on the structure of silica nanoparticles. TEM images of SNP-*4* obtained via changing the amount of ammonium hydroxide into 1.0 mL (A) and 1.8 mL (B).

When the amount of ammonia hydroxide added in the synthesis was 1.0 and 1.8 mL, silica nanoparticles with similar rodlike surface nanotopography were obtained. The particle size / cavity sizes were measured to be ~446 nm and ~ 196 nm at ammonia hydroxide amount of 1.0 mL, and ~ 211 nm and ~ 92 nm at ammonia hydroxide amount of 1.8 mL. It is noted that at the ammonia hydroxide amount of 1.56 mL, silica nanoparticles (SNP-*4*, Fig. 4A_4) with an average particle size of ~ 254 nm and cavity size of ~ 115 nm were obtained (Fig. 3A, Fig. S7). The above observations indicate that the concentration of ammonia hydroxide used in the synthesis is another parameter that can adjust the structure of silica-polymer assembly. Increase in the overall solution alkalinity leads to faster nucleation of APF core with higher numbers and smaller sizes, thus both the particle and cavity sizes are reduced in the resultant silica nanoparticles.

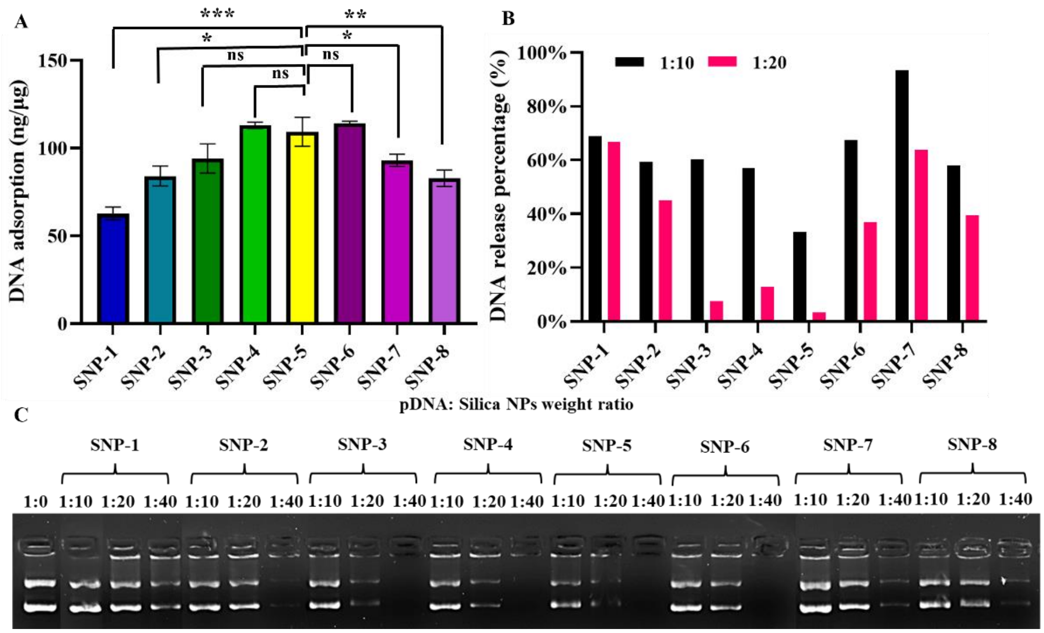

Fig. S21. DNA loading and binding tests. (A) The loading capacity of pDNA-EGFP, (B) quantified pDNA release percentage from (C) via Image J software, and (C) gel-retardation assay of pDNA/SNP-*1~8* with PEI modification at different pDNA/SNP weight ratios. The bars are shown as mean ± SD (n = 3), statistical analysis conducted by t-tests with $p > 0.05$ showing no significant difference (ns), *$p < 0.05$, **$p < 0.01$, ***$p < 0.001$.

The DNA adsorption capability was evaluated via Nanodrop. As shown in Fig. S21A, SNP-*5* showed comparable DNA adsorption ability with SNP-*3, 4, 6* (also with 1D rodlike surface nanotopography), but significantly higher DNA adsorption ability compared with SNP-*7, 8* (with disordered porous structure) and SNP-*1, 2* (with spherical and ellipsoidal topography, respectively). Gel-retardation assay was further performed to study the binding affinity of pDNA by these SNP-*x*. As shown in Fig. S21C, SNP-*3~6* showed a complete DNA binding at pDNA/SNP weight ratio of 1:40. The percentage of unbonded pDNA in all SNP-*x* at weight ratio of pDNA/SNP weight ratio of 1:10 and 1:20 was quantified based on the DNA band intensity through Image J software. As shown in Fig. S21B, SNP-*5* exhibited the lowest DNA release percentage compared with other SNP-*x* at pDNA/SNP weight ratio of 1:10 and 1:20.

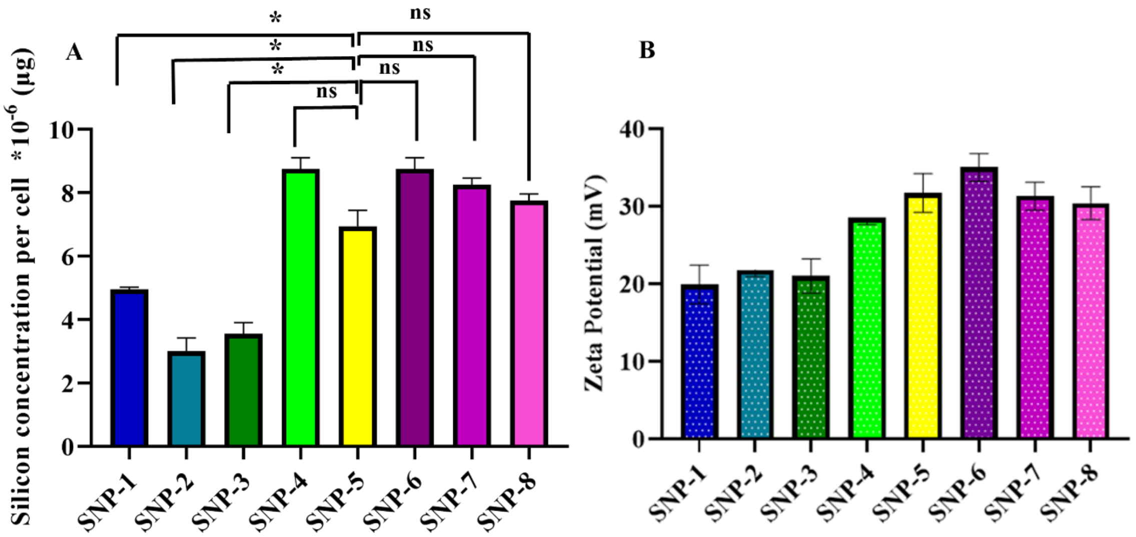

Fig. S22. Cellular uptake and Zeta potential analysis. (A) Cellular uptake of HEK cells incubated with PEI modified SNP-*1~8* loaded with pDNA for 4 h measured by ICP-OES. (B) Zeta potential of PEI modified SNP-*x* measured in water. The bars are shown as mean ± SD (n = 3), statistical analysis conducted by t-tests with $p > 0.05$ showing no significant difference (ns), *$p < 0.05$, **$p < 0.01$, ***$p < 0.001$.

At the same particle concentration of 60 μg/mL, there is no significant difference in the silicon content per cell (Fig. S22A) of SNP-*4, 5, 6, 7, 8*. However their cellular uptake ability was significantly higher than SNP-*1, 2, 3* samples, presumably due to the low specific surface area (< 100 m^2/g , Table S1) of SNP-*1, 2, 3* that are not beneficial for PEI modification and cellular uptake (*6*). As shown in Fig. S22B, the zeta potential value of SNP-*1, 2, 3* (19.9, 21.0 and 21.0 mV, respectively) was generally lower than that of other SNP samples (all above 28.5 mV). Although SNP-*7* and SNP-*8* possess the highest specific surface area (454.9 and 672.7 m^2/g, respectively), their relatively lower zeta potential value compared to SNP-*5* and SNP-6 may be attributed to the ultrasmall pore size (< 2 nm) that is not beneficial for PEI modification.

Table S1. Textual properties of SNP-*x* (x=*1~8*, A_1-A_8)

Sample ID	S (m^2/g)	D (nm)	V (cm^3/g)
**SNP-1**	50.1	-	0.38
**SNP-2**	76.8	-	0.45
**SNP-3**	99.4	-	0.38
**SNP-4**	175.1	50.0	0.67
**SNP-5**	337.7	14.4	0.85
**SNP-6**	430.9	7.5	0.79
**SNP-7**	454.9	1.8-5.6	0.65
**SNP-8**	672.7	1.6	0.41

Note: S is BET specific surface area, D is average pore size estimated from the pore size distribution curves; V is total pore volume.
Type or paste caption here. Create a page break and paste in the Table above the caption.

Table S2. Textual properties of CNP-*x* (x=*1~8*, A_1-A_8)

Sample ID	S (m^2/g)	D (nm)	V (cm^3/g)
**CNP-1**	216.6	-	0.36
**CNP-2**	364.5	50.0	1.00
**CNP-3**	468.9	34.0	1.60
**CNP-4**	664.9	21.0	1.84
**CNP-5**	819.2	8.5	1.23
**CNP-6**	986.8	4.7	1.46
**CNP-7**	1304.2	3.2	1.23
**CNP-8**	547.2	2.4	0.44

Note: S is BET specific surface area, D is average pore size estimated from the pore size distribution curves; V is total pore volume.

Type or paste caption here. Create a page break and paste in the Table above the caption.

REFERENCES AND NOTES

1. G. M. Whitesides, B. Grzybowski, Self-assembly at all scales. *Science* **295**, 2418–2421 (2002).

2. K. Schäfer, H. B. Kolli, M. K. Christensen, S. L. Bore, G. Diezemann, J. Gauss, G. Milano, R. Lund, M. Cascella, Supramolecular packing drives morphological transitions of charged surfactant micelles. *Angew. Chem. Int. Ed. Engl.* **59**, 18591–18598 (2020).

3. D. Danino, Y. Talmon, H. Levy, G. Beinert, R. Zana, Branched threadlike micelles in an aqueous solution of a trimeric surfactant. *Science* **269**, 1420–1421 (1995).

4. S. Jain, F. S. Bates, On the origins of morphological complexity in block copolymer surfactants. *Science* **300**, 460–464 (2003).

5. S. A. Safran, P. Pincus, D. Andelman, Theory of spontaneous vesicle formation in surfactant mixtures. *Science* **248**, 354–356 (1990).

6. C. G. Goltner, M. Antonietti, Mesoporous materials by templating of liquid crystalline phases. *Adv. Mater.* **9**, 431–436 (1997).

7. T. Zhao, A. Elzatahry, X. Li, D. Zhao, Single-micelle-directed synthesis of mesoporous materials. *Nat. Rev. Mater.* **4**, 775–791 (2019).

8. S. Che, A. E. Garcia-Bennett, T. Yokoi, K. Sakamoto, H. Kunieda, O. Terasaki, T. Tatsumi, A novel anionic surfactant templating route for synthesizing mesoporous silica with unique structure. *Nat. Mater.* **2**, 801–805 (2003).

9. Y. Wan, D. Zhao, On the controllable soft-templating approach to mesoporous silicates. *Chem. Rev.* **107**, 2821–2860 (2007).

10. X. Zhang, Y. Li, C. Cao, Facile one-pot synthesis of mesoporous hierarchically structured silica/carbon nanomaterials. *J. Mater. Chem.* **22**, 13918–13921 (2012).

11. W. Stöber, A. Fink, E. Bohn, Controlled growth of monodisperse silica spheres in the micron size range. *J. Colloid Interf. Sci.* **26**, 62–69 (1968).

12. J. Liu, S. Z. Qiao, H. Liu, J. Chen, A. Orpe, D. Y. Zhao, G. Q. M. Lu, Extension of the Stöber method to the preparation of monodisperse resorcinol-formaldehyde resin polymer and carbon spheres. *Angew. Chem. Int. Ed. Engl.* **50**, 5947–5951 (2011).

13. A. H. Lu, G. P. Hao, Q. Sun, Can carbon spheres be created through the stöber method? *Angew. Chem. Int. Ed.* **50**, 9023–9025 (2011).

14. B. D. Credico, L. Vigano, C. Canevali, M. D'Arienzo, S. Mostoni, R. Nistico, R. Scotti, Silica nanoparticles self-assembly process in polymer composites: Towards advanced materials. *Ceram. Int.* **49**, 26165–26181 (2023).

15. J. N. Cha, G. D. Stucky, D. E. Morse, T. J. Deming, Biomimetic synthesis of ordered silica structures mediated by block copolypeptides. *Nature* **403**, 289–292 (2000).

16. K. Nakanishi, N. Tanaka, Sol–gel with phase separation. Hierarchically porous materials optimized for high-performance liquid chromatography separations. *Acc. Chem. Res.* **40**, 863–873 (2007).

17. O. Noonan, H. Zhang, H. Song, C. Xu, X. Huang, C. Yu, In situ stöber templating: Facile synthesis of hollow mesoporous carbon spheres from silica-polymer composites for ultra-high level in-cavity adsorption. *J. Mater. Chem. A* **4**, 9063–9071 (2016).

18. H. W. Zhang, O. Noonan, X. D. Huang, Y. N. Yang, C. Xu, L. Zhou, C. Z. Yu, Surfactant-free assembly of mesoporous carbon hollow spheres with large tunable pore sizes. *ACS Nano* **10**, 4579–4586 (2016).

19. A. B. Fuertes, P. Valle-Vigon, M. Sevilla, One-step synthesis of silica@ resorcinol-formaldehyde spheres and their application for the fabrication of polymer and carbon capsules. *Chem. Commun.* **48**, 6124–6126 (2012).

20. H. Song, Y. Nor, M. Yu, Y. Yang, J. Zhang, H. Zhang, C. Xu, N. Mitter, C. Yu, Silica nanopollens enhance adhesion for long-term bacterial inhibition. *J. Am. Chem. Soc.* **138**, 6455–6462 (2016).

21. H. Song, M. Yu, Y. Lu, Z. Gu, Y. Yang, M. Zhang, J. Y. Fu, C. Yu, Plasmid DNA delivery: Nanotopography matters. *J. Am. Chem. Soc.* **139**, 18247–18254 (2017).

22. J. Fu, Z. Gu, Y. Liu, J. Zhang, H. Song, Y. Yang, Y. Yang, O. Noonan, J. Tang, C. Yu, Bottom-up self-assembly of heterotrimeric nanoparticles and their secondary Janus generations. *Chem. Sci.* **10**, 10388–10394 (2019).

23. Y. Liu, H. Zhang, O. Noonan, C. Xu, Y. Niu, Y. Yang, L. Zhou, X. Huang, C. Yu, Kinetically controlled assembly of nitrogen-doped invaginated carbon nanospheres with tunable mesopores. *Chemistry* **22**, 14962–14967 (2016).

24. H. Zhang, M. Yu, H. Song, O. Noonan, J. Zhang, Y. Yang, L. Zhou, C. Yu, Self-organized mesostructured hollow carbon nanoparticles via a surfactant-free sequential heterogeneous nucleation pathway. *Chem. Mater.* **27**, 6297–6304 (2015).

25. S.-C. Mei, G.-X. Huang, X.-H. Rui, L. Li, M.-K. Ke, X.-Q. Pan, Z.-H. Wang, X.-D. Yang, H.-Q. Yu, Y. Yu, Sequential assembly tailored interior of porous carbon spheres for boosted water decontamination through peroxymonosulfate activation. *Adv. Funct. Mater.* **32**, 2111184 (2022).

26. S. C. Mei, X. H. Rui, L. Li, G. X. Huang, X. Q. Pan, M. K. Ke, Z. H. Wang, H. Q. Yu, Y. Yu, Quantitative coassembly for precise synthesis of mesoporous nanospheres with pore structure-dependent catalytic performance. *Adv. Mater.* **33**, e2103130 (2021).

27. J. N. Israelachvili, D. J. Mitchell, B. W. Ninham, Theory of self-assembly of hydrocarbon amphiphiles into micelles and bilayers. *J. Chem. Soc. Faraday Trans.* **72**, 1525–1568 (1976).

28. G. S. Attard, J. C. Glyde, C. G. Goltner, Liquid-crystalline phases as templates for the synthesis of mesoporous silica. *Nature* **378**, 366–368 (1995).

29. C. T. Kresge, M. E. Leonowicz, W. J. Roth, J. C. Vartuli, J. S. Beck, Ordered mesoporous molecular-sieves synthesized by a liquid-crystal template mechanism. *Nature* **359**, 710–712 (1992).

30. W. Wang, P. Wang, X. Tang, A. A. Elzatahry, S. W. Wang, D. Al-Dahyan, M. Zhao, C. Yao, C.-T. Hung, X. Zhu, T. C. Zhao, X. Li, F. Zhang, D. Zhao, Facile synthesis of uniform virus-like mesoporous silica nanoparticles for enhanced cellular internalization. *ACS Cent. Sci.* **3**, 839–846 (2017).

31. Z. Zhao, Y. Zhao, R. Lin, Y. Ma, L. Wang, L. Liu, K. Lan, J. Zhang, H. Chen, M. Liu, F. Bu, P. Zhang, L. Peng, X. Zhang, Y. Liu, C.-T. Hung, A. Dong, W. Li, D. Zhao, Modular super-assembly of hierarchical superstructures from monomicelle building blocks. *Sci. Adv.* **8**, eabo0283 (2022).

32. Z. Zhao, L. Duan, Y. Zhao, L. Wang, J. Zhang, F. Bu, Z. Sun, T. Zhang, M. Liu, H. Chen, Y. Yang, K. Lan, Z. Lv, L. Zu, P. Zhang, R. Che, Y. Tang, D. Chao, W. Li, D. Zhao, Constructing unique mesoporous carbon superstructures via monomicelle interface confined assembly. *J. Am. Chem. Soc.* **144**, 11767–11777 (2022).

33. H. Li, L. Chen, X. Li, D. Sun, H. Zhang, Recent progress on asymmetric carbon-and silica-based nanomaterials: From synthetic strategies to their applications. *Nanomicro Lett.* **14**, 45 (2022).

34. Y. Yang, M. Zhang, H. Song, C. Yu, Silica-based nanoparticles for biomedical applications: From nanocarriers to biomodulators. *Acc. Chem. Res.* **53**, 1545–1556 (2020).

35. A. Saleem, Y. Z. Zhang, M. Usman, M. Haris, P. Li, Tailored architectures of mesoporous carbon nanostructures: From synthesis to applications. *Nano Today* **46**, 101607 (2022).

36. R. K. Kankala, Y. H. Han, J. Na, C. H. Lee, Z. Q. Sun, S. B. Wang, T. Kimura, Y. S. Ok, Y. Yamauchi, A. Z. Chen, K. C.-W. Wu, Nanoarchitectured structure and surface biofunctionality of mesoporous silica nanoparticles. *Adv. Mater.* **32**, 1907035 (2020).

37. J. W. F. To, Z. Chen, H. B. Yao, J. J. He, K. Kim, H. H. Chou, L. J. Pan, J. Wilcox, Y. Cui, Z. Bao, Ultrahigh surface area three-dimensional porous graphitic carbon from conjugated polymeric molecular framework. *ACS Cent. Sci.* **1**, 68–76 (2015).

38. Y. Han, J. Y. Ying, Generalized fluorocarbon-surfactant-mediated synthesis of nanoparticles with various mesoporous structures. *Angew. Chem. Int. Ed.* **44**, 288–292 (2005).

39. M. Thommes, A. V. Neimark, J. P. Olivier, F. Rodriguez-Reinoso, J. Rouquerol, K. S. W. Sing, Physisorption of gases, with special reference to the evaluation of surface area and pore size distribution (IUPAC Technical Report). *Pure Appl. Chem.* **87**, 1051–1069 (2015).

40. P. Van der Voort, P. I. Ravikovitch, K. P. De Jong, A. V. Neimark, A. H. Janssen, M. Benjelloun, E. Van Bavel, P. Cool, B. M. Weckhuysen, E. F. Vansant, Plugged hexagonal templated silica: A unique micro- and mesoporous composite material with internal silica nanocapsules. *Chem. Commun. (Camb)* **9**, 1010–1011 (2002).

41. H. Song, Y. Yang, J. Geng, Z. Gu, J. Zou, C. Yu, Electron tomography: A unique tool solving intricate hollow nanostructures. *Adv. Mater.* **31**, 1801564 (2019).

42. R. Leary, P. A. Midgley, J. M. Thomas, Recent advances in the application of electron tomography to materials chemistry. *Acc. Chem. Res.* **45**, 1782–1791 (2012).

43. D. J. De Rosier, A. Klug, Reconstruction of three dimensional structures from electron micrographs. *Nature* **217**, 130–134 (1968).

44. S. L. J. Thomä, S. W. Krauss, M. Eckardt, P. Chater, M. Zobel, Atomic insight into hydration shells around facetted nanoparticles. *Nat. Commun.* **10**, 995 (2019).

45. N. P. Wickramaratne, J. T. Xu, M. Wang, L. Zhu, L. M. Dai, M. Jaroniec, Nitrogen enriched porous carbon spheres: Attractive materials for supercapacitor electrodes and CO_2 adsorption. *Chem. Mater.* **26**, 2820–2828 (2014).

46. X. H Cui, S. Mao, M. Liu, H. Yuan, Y. Du, Mechanism of surfactant micelle formation. *Langmuir* **24**, 10771–10775 (2008).

47. S. Ghosh, A. Ray, N. Pramanik, Self-assembly of surfactants: An overview on general aspects of amphiphiles. *Biophys. Chem.* **265**, 106429 (2020).

48. C. C. M. C. Carcouët, M. W. P. van de Put, B. Mezari, P. C. M. M. Magusin, J. Laven, P. H. H. Bomans, H. Friedrich, A. C. C. Esteves, N. A. J. M. Sommerdijk, R. A. T. M. van Benthem, G. de With, Nucleation and growth of monodisperse silica nanoparticles. *Nano Lett.* **14**, 1433–1438 (2014).

49. Y. Han, Z. Lu, Z. Teng, J. Liang, Z. Guo, D. Wang, M.-Y. Han, W. Yang, Unraveling the growth mechanism of silica particles in the stöber method: In situ seeded growth model. *Langmuir* **33**, 5879–5890 (2017).

50. D. L. Green, S. Jayasundara, Y. F. Lam, M. T. Harris, Chemical reaction kinetics leading to the first Stober silica nanoparticles – NMR and SAXS investigation. *J. Non Cryst. Solids* **315**, 166–179 (2003).

51. T. Dwars, E. Paetzold, G. Oehme, Reactions in micellar systems. *Angew. Chem. Int. Ed. Engl.* **44**, 7174–7199 (2005).

52. A. Sorrenti, O. Illa, R. M. Ortuno, Amphiphiles in aqueous solution: Well beyond a soap bubble. *Chem. Soc. Rev.* **42**, 8200–8219 (2013).

53. A. E. C. Palmqvist, Synthesis of ordered mesoporous materials using surfactant liquid crystals or micellar solutions. *Curr. Opin. Colloid Interface Sci.* **8**, 145–155 (2003).

54. C.-F. Cheng, Z. Luan, J. Klinowski, The role of surfactant micelles in the synthesis of the mesoporous molecular-sieve MCM-41. *Langmuir* **11**, 2815–2819 (1995).

55. P. Innocenzi, L. Malfatti, T. Kldchob, P. Falcaro, Order-disorder in self-assembled mesostructured silica films: A concepts review. *Chem. Mater.* **21**, 2555–2564 (2009).

56. C. Xu, C. Lei, Y. Wang, C. Yu, Dendritic mesoporous nanoparticles: Structure, synthesis and properties. *Angew. Chem. Int. Ed. Engl.* **61**, e202112752 (2022).

57. Y. Wang, B. Zhang, X. Ding, X. Du, Dendritic mesoporous organosilica nanoparticles (DMONs): Chemical composition, structural architecture, and promising applications. *Nano Today* **39**, 101231 (2021).

58. K. L. Cao, A. F. Arif, K. Kamikubo, T. Izawa, H. Iwasaki, T. Ogi, Controllable synthesis of carbon-coated SiO_x particles through a simultaneous reaction between the hydrolysis-condensation of tetramethyl orthosilicate and the polymerization of 3-aminophenol. *Langmuir* **35**, 13681–13692 (2019).

59. J. Fu, J. Jiao, H. Song, Z. Gu, Y. Liu, J. Geng, K. S. Jack, A. Du, J. Tang, C. Yu, Fractal-in-a-sphere: Confined self-assembly of fractal silica nanoparticles. *Chem. Mater.* **32**, 341–347 (2020).

60. L. Xie, M. Yan, T. Liu, K. Gong, X. Luo, B. Qiu, J. Zeng, Q. Liang, S. Zhou, Y. He, W. Zhang, Y. Jiang, Y. Yu, J. Tang, K. Liang, D. Zhao, B. Kong, Kinetics-controlled super-assembly of asymmetric

porous and hollow carbon nanoparticles as light-sensitive smart nanovehicles. *J. Am. Chem. Soc.* **144**, 1634–1646 (2022).

61. M. Gulumian, C. Andraos, A. Afantitis, T. Puzyn, N. J. Coville, Importance of surface topography in both biological activity and catalysis of nanomaterials: Can catalysis by design guide safe by design? *Int. J. Mol. Sci.* **22**, 8347 (2021).

62. R. K. Singh, J. C. Knowles, H.-W. Kim, Advances in nanoparticle development for improved therapeutics delivery: Nanoscale topographical aspect. *J. Tissue. Eng.* **10**, 2041731419877528 (2019).

63. J. A. Finbloom, C. Huynh, X. Huang, T. A. Desai, Bioinspired nanotopographical design of drug delivery systems. *Nat. Rev. Bioeng.* **1**, 139–152 (2023).

64. D. Cheng, S. Theivendran, J. Tang, L. Cai, J. Zhang, H. Song, C. Yu, Surface chemistry of spiky silica nanoparticles tailors polyethyleneimine binding and intracellular DNA delivery. *J. Colloid Interf. Sci.* **628**, 297–305 (2022).

65. J. Zhao, W. Niu, L. Zhang, H. Cai, M. Han, Y. Yuan, S. Majeed, S. Anjum, G. Xu, A template-free and surfactant-free method for high-yield synthesis of highly monodisperse 3-aminophenol-formaldehyde resin and carbon nano/microspheres. *Macromolecules* **46**, 140–145 (2013).
